# Efficient increase of ɣ-aminobutyric acid (GABA) content in tomato fruits by targeted mutagenesis

**DOI:** 10.1038/s41598-017-06400-y

**Published:** 2017-08-01

**Authors:** Satoko Nonaka, Chikako Arai, Mariko Takayama, Chiaki Matsukura, Hiroshi Ezura

**Affiliations:** 1 Faculty of Life and Environmental Sciences, University of Tsukuba, 1-1-1 Tennodai, Tsukuba Ibaraki, 305-8572 Japan; 2 Gene Research Center, University of Tsukuba, 1-1-1 Tennodai, Tsukuba Ibaraki, 305-8572 Japan; 3 Graduate School of Life and Environmental Sciences, University of Tsukuba, 1-1-1 Tennodai, Tsukuba Ibaraki, 305-8572 Japan

**Keywords:** Molecular engineering in plants, Molecular engineering in plants, Plant breeding, Plant breeding

## Abstract

γ-Aminobutyric acid (GABA) is a non-proteinogenic amino acid that has hypotensive effects. Tomato (*Solanum lycopersicum* L.) is among the most widely cultivated and consumed vegetables in the world and contains higher levels of GABA than other major crops. Increasing these levels can further enhance the blood pressure-lowering function of tomato fruit. Glutamate decarboxylase (GAD) is a key enzyme in GABA biosynthesis; it has a C-terminal autoinhibitory domain that regulates enzymatic function, and deleting this domain increases GAD activity. The tomato genome has five GAD genes (*SlGAD1*–*5*), of which two (*SlGAD2* and *SlGAD3*) are expressed during tomato fruit development. To increase GABA content in tomato, we deleted the autoinhibitory domain of *SlGAD2* and *SlGAD3* using clustered regularly interspaced short palindromic repeats (CRISPR)/CRISPR-associated protein (Cas)9 technology. Introducing a stop codon immediately before the autoinhibitory domain increased GABA accumulation by 7 to 15 fold while having variable effects on plant and fruit size and yield. This is the first study describing the application of the CRISPR/Cas9 system to increase GABA content in tomato fruits. Our findings provide a basis for the improvement of other types of crop by CRISPR/Cas9-based genetic modification.

## Introduction

Lifestyle is closely related to human health. Diet, physical activity, stress, smoking, and drinking are the major causes of diseases such as high blood pressure, diabetes, hyperlipidaemia, and obesity. High blood pressure is a risk factor for heart diseases and stroke, both of which are leading causes of death worldwide. In addition, as the estimated number of patients with high blood pressure reached 1.13 billion in 2015^1^, its prevention and control are globally required.

The ɣ-aminobutyric acid (GABA) is a non-proteinogenic amino acid, which is widely found in bacteria, animals, and plants. Several studies in humans and experimental animals have shown that administration of exogenous GABA is effective in lowering blood pressure of hypertensive patients but not of normotensive one^2–4^. Since dietary therapy is used more preferentially than medication as an initial treatment for patients with mild high blood pressure or high-normal blood pressure, intake of GABA through daily diet may help reduce their symptoms. To this end, many have attempted to increase GABA content in a variety of local foods, such as tea, rice and fermented foods, (*i.e*. fermented milk, tempeh, yogurt and soy sauce)^5–9^. As these GABA-enriched products brought anti-hypotensive effects in experimental animals or human^7, 9–11^, daily intake of GABA-enriched foods would be an effective way to prevent hypertension.

To further promote the consumption of GABA-enriched foods in daily life, the selection of materials for enrichment of GABA may be important, because food culture generally differs among countries. It is considered that the ideal material is such a thing consumed worldwide and has high potential to produce GABA. Tomato is one of the most produced vegetables in the world and widely consumed in daily diet. In addition, tomato contains relatively higher levels of GABA than other major crops^12^, probably due to its higher productivity and/or capacity to store GABA. In cultivated tomato, GABA is intensively accumulated in green-stage fruits (comprising up to 50% of the total free amino acids), but when fruits reach the red stage, GABA levels decline to less than 20% of the total free amino acids^13, 14^. To understand the molecular mechanisms underlying the changes in GABA accumulation in tomato fruits, we previously isolated GABA metabolism-related genes from tomato cultivar ‘Micro-Tom’^15^. In higher plants, GABA is predominantly metabolized through a pathway called the ‘GABA shunt’, which partially bypasses the tricarboxylic acid (TCA) cycle^16^. In this pathway, GABA is biosynthesized from glutamate by glutamate decarboxylase (GAD), and then catabolized to succinate by GABA transaminase (GABA-T) and succinic semialdehyde dehydrogenase (SSADH) in sequential reactions. We found that at least three GAD genes (*SlGAD1–3*), three GABA-T genes (*SlGABA-T1–3*), and one SSADH gene (*SlSSADH*) are expressed during tomato fruit development^17^. Loss of function analyses revealed that the expression of *SlGAD2* and/or *SlGAD3*, and *SlGABA-T1* is important for GABA biosynthesis and catabolism, respectively^14, 18^. Thus, GABA levels in tomato can be increased by genetically manipulating *SlGAD2, SlGAD3* or *SlGABA-T1* expression. For example, when *SlGAD3* was overexpressed using the tomato fruit ripening-specific E8 promoter/*Arabidopsis* heat shock protein (HSP) 18.2 terminator expression cassette, the GABA level of red-stage fruits increased to up to six- or seven-fold that of wild-type (WT) tomato^19^. On the other hand, when *SlGABA-T1* expression was suppressed by RNA interference under the control of the cauliflower mosaic virus (CaMV) 35S promoter/nopaline synthase terminator expression cassette, the GABA level in red-stage fruits increased to up to 9.2-folds that of WT tomato^14^. It is noteworthy that the fruit specific overexpression of a mutant *SlGAD3*, in which 87 C-terminal nucleotides (corresponding to 29 amino acids) were deleted, was even more effective in increasing the GABA level (11- to 18-folds that of WT red-stage tomato)^19^. This was likely the consequence of an increased GAD activity because plant GADs generally have an autoinhibitory domain at the C-terminus. This domain is composed of 30–50 amino acids and, in many cases, it also functions as the calmodulin (CaM)-binding domain (CaMBD). It is considered that this domain inhibits GAD activity at the physiological pH by folding its active site, and this autoinhibition is suppressed when the active site is unfolded by conformational changes via an acidic pH or by binding of Ca^2+^/CaM to the CaMBD at the physiological pH^20^. Thus, it is assumed that removal of this domain allows the enzyme to exert constitutive activity, resulting in high GABA accumulation. As enhanced GABA productivity by C-terminal truncation of GAD has been reported in several plant species^21–23^, this trait would be widely applicable for improving GABA accumulation in plant cells.

Concerning the development of breeding materials, the application of mutagenesis has been more acceptable than that of transgenesis. Thus, we previously attempted to isolate *SlGAD3* mutants from a ‘Micro-Tom’ ethyl methanesulfonate (EMS) mutant population^15^. Although approximately 4,500 lines of EMS mutant alleles were screened by Targeting Induced Local Lesions In Genomes (TILLING), an efficient tool for screening point mutations^24^, no mutation was found around any C-terminal region of *SlGAD3* (Ezura *et al*., unpublished results).

The Clustered, Regularly Interspaced, Short Palindromic Repeats/CRISPR-associated protein 9 (CRISPR/Cas9) system is a powerful tool for targeted mutagenesis in various organisms^25–28^. This system allows inducing double-strand breaks (DSBs) at a specific genome region and mutations, preferentially via the error-prone non-homologous end-joining (NHEJ) pathway. Although it requires designing a 20 bp-target sequence at the genome region before the protospacer-adjacent motif (PAM) sequence (5′-NGG for the type II CRISPR/Cas9 system)^29^, the advantages with respect to ease of construction and high mutagenesis efficiency have accelerated the use of this system in various areas of research. In tomato, the CRISPR/Cas9 system has also been applied to successfully induce mutations at target sites^30–32^.

In the present study, we induced mutations at the C-terminal region of two tomato GAD genes (*SlGAD2* and *SlGAD3*) utilizing the CRISPR/Cas9 system to produce GABA-enhanced tomato fruits. Insertions and deletions were frequently detected at the target sites of the *SlGAD2-* and *SlGAD3-*targeted lines. In addition, the GABA content was greatly increased in the leaves and red-stage fruits of some mutant lines carrying the mutant allele that produced the GAD protein without the C-terminal domain.

## Results

### CRISPR/Cas9 target sites in SlGAD2 and SlGAD3

**Figure 1:**
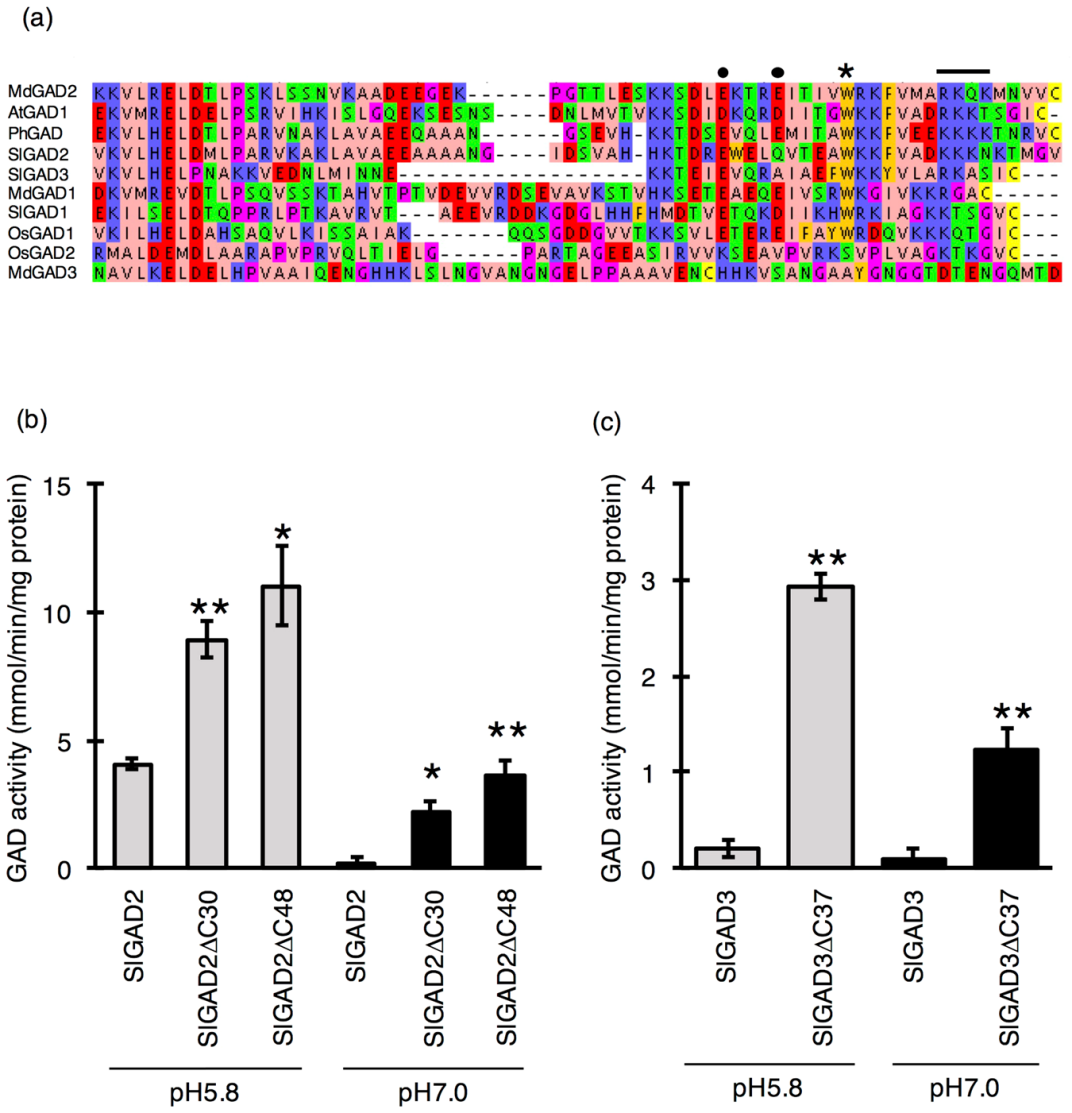
Determination of the target sites within SlGADs’ C-terminal regions. (**a**) Multiple sequence alignment of the C-terminal region of 10 plant GADs. The C-terminal of MdGAD1-3, AtGAD1, PhGAD, OsGAD1-2, SlGAD1-3 from *Malus domestica* (apple), *Arabidopsis thaliana*, *Oryza sativa* (rice), and *Solanum lycopersicum* (tomato), respectively, which were obtained in a previous study, were compared. The conserved tryptophan (W) residue and lysine (K) clusters involved in CaM-binding^19, 48^ are indicated by an asterisk and a black line, respectively. The two glutamate (E) residues functioned as pseudo-substrates and are likely involved in autoinhibition in PhGAD^43^ are indicated as black circle. Accession Numbers: MdGAD1, KC812242; MdGAD2, KC812243; MdGAD3, KC812244; AtGAD1, AY094464.1; AtGAD2, U49937.1; PhGAD, L16977; SlGAD1, AB359913; SlGAD2, AV359914.1; SlGAD3, AB359915.1; OsGAD1, AB056060; OsGAD2; AB056061. (**b** and **c**) GAD activity of SlGAD2ΔC30 and SlGAD2ΔC48, and SlGAD3Δ37, respectively, in *in vitro* assays. Crude extracts from *Escherichia coli* were used in GAD enzymatic assays and Ca^2+^ was not included in reaction buffers. SlGAD2 and SlGAD3 are full-length GADs, whereas SlGAD2ΔC30, SlGAD2ΔC48, and SlGAD3ΔC37 are truncated GADs at C-terminal positions 30, 48, and 37, respectively. Bars represent the standard deviation (n = 3) and asterisks indicate statistical differences in according to the Tukey-Kramer’s test between full-length GADs (*P < 0.05 and **P < 0.01).

To breed a tomato line with high GABA content, the C-terminal regions of SlGAD2 and SlGAD3 were targeted by CRISPR/Cas9 technology. To select target sequence, we compared 10 amino acid sequences from five plant species (Fig. 1a). The 30 and 48 amino acids at the C terminus as target sites were selected for SlGAD2. In SlGAD3, the 37 amino acid was used as a target for CRISPR/Cas9. We used an *Escherichia coli* protein expression system to evaluate the activity of these three SlGADs (referred to as SlGAD2ΔC30, SlGAD2ΔC48, and SlGAD3ΔC37) at pH 5.8 (the optimal value for GAD activity) and pH 7.0 (the pH in plant cells) (Fig. 1b). C-terminal truncation increased SlGAD activity at pH 5.8 and 7.0 in the absence of Ca^2+^/CaM, which is an enhancer of GAD activity (Fig. 1b). SlGAD2ΔC30, SlGAD2ΔC48, and SlGAD3ΔC37 activity was 2.2, 2.7, and 14.6 times higher, respectively, at pH 5.8 and 10.2, 16.5, and 12.9 times higher, respectively, at pH 7.0 than that of full-length SlGADs. Based on these results, we predicted that inducing the stop codon at 30 and 48 amino acids for SlGAD2 and 37 amino acids for SlGAD3 via CRISPR/Cas9 would lead to increased GABA accumulation in tomato fruits.

### Selection of the T_0_ lines with mutated target sites

According to above result, we constructed CRISPR/Cas9 vectors (TG2C30, TG2C48, and TG3C37). These vectors were introduced into tomato via *Agrobacterium*-mediated transformation^33^. Fourteen, 16, and 35 tomato lines with single or double copies of transgenes were found for TG2C30, TG2C48, and TG3C37, respectively (Supplementary Fig. 1). To detect the mutation, the target site on each transgenic line was cloned into a pGEM-T vector (Promega, Madison, WI, USA) and the resulting 257 sequence clones were checked by Sanger sequencing. Data was summarized in Table 1. Transgenic TG2C30, TG2C48, and TG3C37 lines contained 11, 10, and 9 mutation lines, respectively and each target site contained 6, 14, and 10 types of mutation, respectively. Only one of the 31 lines sequenced (TG2C48 #4) lacked mutations (3.33%; 1/31); the remaining lines presented mutations 3- or 4-bp upstream of PAM. Most (59.9%, 154/257) of the sequenced clones presented deletions ranging from a single to more than 200 nucleotides and 18.3% (47/257) insertions of a single nucleotide; simultaneous insertions and deletions reached 1.2% (3/257), and WT genotypes reached 20.6% (53/257). The most inserted nucleotide was T (55.3%), followed by A (29.8%), G (12.8%), and C (2.1%). The zygosity of the 31 T_0_ plants was predicted from clone sequences (Table 1

**Table 1:** CRISPR/Cas9-induced mutations in target genes.

Line	T_0_		T_1_	
	Zygosity	Genotype	Mutation segregation	T-DNA
*TG2C30*				
#17	Bi-allele	5d50, 2d3	2d50, 2d3	2+: 2−
#9	Chimera	4WT, 2d3, 1i1(A)	13WT	9+: 4−
#13	Chimera	4WT, 2d1, 1d3	17WT	13+: 4−
#24	Homozygote	10d3	13d3	10+: 3−
#14	Homozygote	8d1	16d1	16+
#27	Homozygote	12d4	12 h	11+: 1−
#10	Bi-allele	6d3, 2d1	NT	NT
#4	Homozygote	9d4	NT	NT
#5	Homozygote	9d4	NT	NT
#7	Bi-allele	4d3, 3d2	NT	NT
#18	Bi-allele	3d3, 3i1(A)	NT	NT
			Total homozygote plant (-CRISPR/Cas9)	33 (5)
*TG2C48*				
#56	Chimera	4WT, 2d1, 1d5, 1i1(G)	4i1(G), 1d27, 13 h, 6WT	19+: 11−
#57	Chimera	3d43, 2d2, 1d7, 1i1(A)	13d43, 2i1(G), 1i1(C), 15 h	27+: 11−
#13	Hetero	11WT, 1i1(G)	5d1, 2d7, 1i1(G), 6 h, 1WT	8+: 10−
#10	Bi-allele	7d254, 3d6	6d254, 4d6, 6 h	15+: 3−
#17	Chimera	2WT, 2d1^a^, 1d1^b^, 1i1(C)	NT	NT
#12	Chimera	10WT, 1d3, 1i1(T)	NT	NT
#4	WT	12WT	NT	NT
#7	Chimera	4d66, 3d12, 3d18, 3d48	NT	NT
#11	Bi-allele	5d2, 3d1	NT	NT
#33	Hetero	2WT, 1i1(T)	NT	NT
#52	Chimera	4WT, 3i1(G), 2d9	NT	NT
			Total homozygote plant (-CRISPR/Cas9)	39 (13)
*TG3C37*				
#21	Chimera	4d53, 4i1(T), 1d11	4i1(T), 3d53, 17 h	15+: 11−
#3	Bi-allele	6d4, 2(d6i1)	4d4, 3(d6i1), 13 h	12+: 9−
#4	Bi-allele	9i1(A), 2i1(T)	7i1(T), 6i1(A), 6 h	15+: 8−
#6	Bi-allele	4i1(T), 1i1(G)	5i1(G), 3i1(T), 11 h	19+: 1−
#15	Chimera	6i1(T), 1d1i1, 1d27	NT	NT
#10	Bi-allele	4d3, 3i1(T)	NT	NT
#8	Bi-allele	5i1(T), 3d3	NT	NT
#23	Bi-allele	2d6, 2d123	NT	NT
#9	Bi-allele	4d6, 3d3	NT	NT
			Total homozygote plant (-CRISPR/Cas9)	35 (12)

The zygosities, such as homozygote, bi-allele and heterozygote, were estimated on T_0_ plant. WT, wild-type sequence with no mutation detected. “d” and “i” mean deletion and insertion, respectively. (A,T,G,C) after “i” indicated the inserted base pair. “il (T) means “T” was inserted on target site.

In T_0_ generation, “d#” or “i#” is # of base pair were deleted or were inserted on target site. Numbers before “d” or “i” mean the number of clones we checked. “d1i1” means 1 bp was deleted and inserted at the same time.

“a” and “b” in Superscript mean different type of 1 base pair deletion. In T_1_ generation, numbers before “d” or “i” means plant number with mutation in each lines. All “d” and “i” are homozygous. “h” is heterozygous. “NT” means not-tested. “+” and “−” are with or without T-DNA insertion. The number before “+” or “−” is plant numbers.

), and the alleles of the target site were classified into four groups: if only one type of mutation was detected, lines were presumed to be homozygous (16.1%, 5/31); if there were two types of mutation, they were considered as bi-allelic (41.9%, 13/31); if a line had one type of mutation and a WT allele, it was classified as heterozygous (6.5%, 2/31); and if more than three types of mutation and the WT were present, we considered that the line was chimeric (32.3%, 10/31). If no mutation was detected, then the line was presumed to be WT (3.2%, 1/31).

### Characterization of the mutations induced at the C-Terminal region of T_0_

To verify the introduction of a stop codon immediately upstream of autoinhibitory domain, its nucleic acid sequence was translated to the corresponding amino acid sequence. Mutant lines were thus classified into four types, depending on their C-terminal amino acid sequence (Table 2, Supplementary Fig. 2): Type 1 had a stop codon immediately upstream of the autoinhibitory domain (i.e., they lacked a C-terminal); Type 2 had a conserved or defective autoinhibitory C-terminal domain, and the size of the C-terminal was almost identical to that of WT; Type 3 contained both Type 1 and Type 2 C-terminals; and Type 4 contained terminals that were both remarkably short and identical to WT C-terminals (Supplementary Fig. 2

**Table 2:** Amino acid sequence of Target site (T0 generation).

Line	Genotype	Amino acid sequence		
GAD2	WT	**DIV(10aa)RVKAKLAVAEEAAAANGIDSVAHH***K*TDREWELQVTEAWKKFVADKKKNKTMGVC*	Type of C-terminal	GABA
*TG2C30*				
#17	d3	**DIV(10aa)RVKAKLAVAEEAAAANGIDSVAHH**-TDREWELQVTEAWKKFVADKKKNKTMGVC*	3	Higher
	d50	**DIV(10aa)RVKAKLAVAEEAAAANGIDSV**EEVCC*		
#9	WT	**DIV(10aa)RVKAKLAVAEEAAAANGIDSVAHH***K*TDREWELQVTEAWKKFVADKKKNKTMGVC*	3	Higher
	d3	**DIV(10aa)RVKAKLAVAEEAAAANGIDSVAH**Q-TDREWELQVTEAWKKFVADKKKNKTMGVC*		
	i1(A)	**DIV(10aa)RVKAKLAVAEEAAAANGIDSVAHHK**NG*		
#13	WT	**DIV(10aa)RVKAKLAVAEEAAAANGIDSVAHH***K*TDREWELQVTEAWKKFVADKKKNKTMGVC*	2	Higher
	d3	**DIV(10aa)RVKAKLAVAEEAAAANGIDSVAHH**-TDREWELQVTEAWKKFVADKKKNKTMGVC*		
	d1	**DIV(10aa)RVKAKLAVAEEAAAANGIDSVAHH***K*RIENGSYRLLKHGRSLLLIKRRIRLWEFVN*		
#24	d3	**DIV(10aa)RVKAKLAVAEEAAAANGIDSVAH**Q-TDREWELQVTEAWKKFVADKKKNKTMGVC*	2	Same
#10	d3	**DIV(10aa)RVKAKLAVAEEAAAANGIDSVAH**Q-TDREWELQVTEAWKKFVADKKKNKTMGVC*	2	Same
	d1	**DIV(10aa)RVKAKLAVAEEAAAANGIDSVAHH***K*RIENGSYRLLKHGRSLLLIKRRIRLWEFVN*		
#14	d1	**DIV(10aa)RVKAKLAVAEEAAAANGIDSVAHH***K*RIENGSYRLLKHGRSLLLIKRRIRLWEFVN*	2	Same
#27	d4	**DIV(10aa)RVKAKLAVAEEAAAANGIDSVAH**-*K*RIENGSYRLLKHGRSLLLIKRRIRLWEFVN*	2	Same
#4	d4	**DIV(10aa)RVKAKLAVAEEAAAANGIDSVAH**-*K*RIENGSYRLLKHGRSLLLIKRRIRLWEFVN*	2	Same
#5	d4	**DIV(10aa)RVKAKLAVAEEAAAANGIDSVAH**-*K*RIENGSYRLLKHGRSLLLIKRRIRLWEFVN*	2	N.D.
#7	d3	**DIV(10aa)RVKAKLAVAEEAAAANGIDSVAH**-*K*TDREWELQVTEAWKKFVADKKKNKTMGVC*	3	N.D.
	d2	**DIV(10aa)RVKAKLAVAEEAAAANGIDSVAH**QNG*		
#18	d3	**DIV(10aa)RVKAKLAVAEEAAAANGIDSVAH**Q-TDREWELQVTEAWKKFVADKKKNKTMGVC*	3	N.D.
	i1(A)	**DIV(10aa)RVKAKLAVAEEAAAANGIDSVAHH***K*NG*		
GAD2	WT	**DIV(10aa)RVKAKL***A*VAEEAAAANGIDSVAHHKTDREWELQVTEAWKKFVADKKKNKTMGVC*	Type of C-terminal	GABA
*TG2C48*				
#56	WT	**DIV(10aa)RVKAKL***A*VAEEAAAANGIDSVAHHKTDREWELQVTEAWKKFVADKKKNKTMGVC*	3	Higher
	d1	**DIV(10aa)RVKAKL**PWPRRRPRRTVSTAWHIIKRIENGSYRLLKHGRSLLLIKRRIRLWEFVN*		
	i1(G)	**DIV(10aa)RVKAKL**GRGRGGGRGERYRQRGTS*		
	d5	**DIV(10aa)RVKASR**GRGGGRGERYRQRGTS*		
#57	d43	**DIV(10aa)R**TVSTAWHIIKRIENGSYRLLKHGRSLLLIKRRIRLWEFVN*	3	Higher
	d7	**DIV(10aa)RVKA**PWPRRRPRRTVSTAWHIIKRIENGSYRLLKHGRSLLLIKRRIRLWEFVN*		
	d2	**DIV(10aa)RVKA**KLRGRGGGRGERYRQRGTS*		
	i1(A)	**DIV(10aa)RVKA**KLDRGRGGGRGERYRQRGTS*		
#13	WT	**DIV(10aa)RVKAKL***A*VAEEAAAANGIDSVAHHKTDREWELQVTEAWKKFVADKKKNKTMGVC*	3	Higher
	i1(G)	**DIV(10aa)RVKAKL**GRGRGGGRGERYRQRGTS*		
#17	WT	**DIV(10aa)RVKAKL***A*VAEEAAAANGIDSVAHHKTDREWELQVTEAWKKFVADKKKNKTMGVC*	3	Higher
	d1^a^	**DIV(10aa)RVKAKL**PWPRRRPRRTVSTAWHIIKRIENGSYRLLKHGRSLLLIKRRIRLWEFVN*		
	d1^b^	**DIV(10aa)RVKAKL***A*WPRRRPRRTVSTAWHIIKRIENGSYRLLKHGRSLLLIKRRIRLWEFVN*		
	i1(C)	**DIV(10aa)RVKAKL***A*RGRGGGRGERYRQRGTS*		
#12	WT	**DIV(10aa)RVKAKL***A*VAEEAAAANGIDSVAHHKTDREWELQVTEAWKKFVADKKKNKTMGVC*	3	Higher
	d3	**DIV(10aa)RVKAKP**-VAEEAAAANGIDSVAHHKTDREWELQVTEAWKKFVADKKKNKTMGVC*		
	i1(T)	**DIV(10aa)RVKAK**LVRGRGGGRGERYRQRGTS*		
#10	d6	**DIV(10aa)RVKA**--TVAEEAAAANGIDSVAHHKTDREWELQVTEAWKKFVADKKKNKTMGVC*	4	Same
	d254	DIV*		
#4	WT	**DIV(10aa)RVKAKL***A*VAEEAAAANGIDSVAHHKTDREWELQVTEAWKKFVADKKKNKTMGVC*	—	Same
#7	d12	**DIV**--(13aa)----TVAEEAAAANGIDSVAHHKTDREWELQVTEAWKKFVADKKKNKTMGVC*	2	Same
	d18	**DIV(10aa)RVKAK**------AAAANGIDSVAHHKTDREWELQVTEAWKKFVADKKKNKTMGVC*		
	d48	**DIV-6AA**-------------AAAANGIDSVAHHKTDREWELQVTEAWKKFVADKKKNKTMGVC*		
	d66	**DIV**KVLHELD-----------------NSVAHHKTDREWELQVTEAWKKFVADKKKNKTMGVC*		
#11	d1	**DIV(10aa)RVKAKL**PWPRRRPRRTVSTAWHIIKRIENGSYRLLKHGRSLLLIKRRIRLWEFVN*	3	n.d.
	d2	**DIV(10aa)RVKAKL**RGRGGGRGERYRQRGTS*		
#33	WT	**DIV(10aa)RVKAKL***A*VAEEAAAANGIDSVAHHKTDREWELQVTEAWKKFVADKKKNKTMGVC*	3	n.d.
	i1(T)	**DIV(10aa)RVKAKL**GRGRGGGRGERYRQRGTS*		
#52	WT	**DIV(10aa)RVKAKL***A*VAEEAAAANGIDSVAHHKTDREWELQVTEAWKKFVADKKKNKTMGVC*	3	n.d.
	i1(G)	**DIV(10aa)RVKAKL**GRGRGGGRGERYRQRGTS*		
	d9	**DIV(10aa)RVKA**---VAEEAAAANGIDSVAHHKTDREWELQVTEAWKKFVADKKKNKTMGVC*		
GAD3	WT	**TLADRLVSDIVKVLHELPNAKK***V*EDNLMINNEKKTEIEVQRAIAEFWKKYVLARKASIC*	Type of C-terminal	GABA
*TG3C37*				
#21	i1(T)	**TLADRLVSDIVKVLHELPNAKK***V*GG*	1	Higher
	d11	TLADRLVSDIVKVLHELPNAK*		
	d53	TLADRLVSDIVKVLHELPN*		
#3	d4	**TLADRLVSDIVKVLHELPNAK**WRII*	1	Higher
	d5	**TLADRLVSDIVKVLHELPNA**IGG*		
#4	i1(A)	**TLADRLVSDIVKVLHELPNAKK**DGG*	1	Higher
	i1(T)	**TLADRLVSDIVKVLHELPNAKK***V*GG*		
#6	i1(T)	**TLADRLVSDIVKVLHELPNAKK***V*GG*	1	Higher
	i1(G)	**TLADRLVSDIVKVLHELPNAKK***V*GG*		
#15	d1+i1	**TLADRLVSDIVKVLHELPNAKK**LEDNLMINNEKKTEIEVQRAIAEFWKKYVLARKASIC*	3	Higher
	d27	**TLADRLVSDIVKVLHELPNA**---------NNEKKTEIEVQRAIAEFWKKYVLARKASIC*		
	i1(T)	**TLADRLVSDIVKVLHELPNAKK***V*GG*		
#10	d3	**TLADRLVSDIVKVLHELPNAK**-MEDNLMINNEKKTEIEVQRAIAEFWKKYVLARKASIC*	3	Higher
	i1(T)	**TLADRLVSDIVKVLHELPNAKK***V*GG*		
#8	d3	**TLADRLVSDIVKVLHELPNAK**-*V*EDNLMINNEKKTEIEVQRAIAEFWKKYVLARKASIC*	3	Higher
	i1(T)	**TLADRLVSDIVKVLHELPNAKK***V*GG*		
#23	d6	**TLADRLVSDIVKVLHELPNAK**--EDNLMINNEKKTEIEVQRAIAEFWKKYVLARKASIC*	4	Same
	d123	**TLADR**WKKYVLARKASIC*		
#9	d3	**TLADRLVSDIVKVLHELPNAK**-MEDNLMINNEKKTEIEVQRAIAEFWKKYVLARKASIC*	2	Same
	d6	**TLADRLVSDIVKVLHELPNA**--MEDNLMINNEKKTEIEVQRAIAEFWKKYVLARKASIC*		

WT, wild-type sequence with no mutation detected. “d” and “i” means deletion and insertion, respectively. “*” was stop codon. The same sequence as Wild Type is expressed in **Bold**. The changed sequence by the mutation via CRISPR/Cas9 was in Regular. **Bold with underline** is the putative auto inhibitory domain and CaMd domain. The target site is in *Italic*. “a” and “b” in Superscript mean different one base-pair deletion. “Higher” showed higher GABA content comparing with WT. “Same level” indicated almost the same contain level as WT. n.d. means no data. GABA content was measured using fruits at the Br +10 stage (red).

). Type 3 mutants were the most frequent (15/31 lines), followed by Type 2 (9/31 lines), Type 1 (4/31 lines), and Type 4 (2/31 lines), which were the rarest. Type frequency varied according to target site: Type 2 was most frequent in TG2C30 (7/11 lines), Type 3 in TG2C48 (8/11 lines), and Type 1 in TG3C37 (4/9 lines).

### Evaluation of GABA accumulation in red stage fruit of T_0_ mutant lines

**Figure 2:**
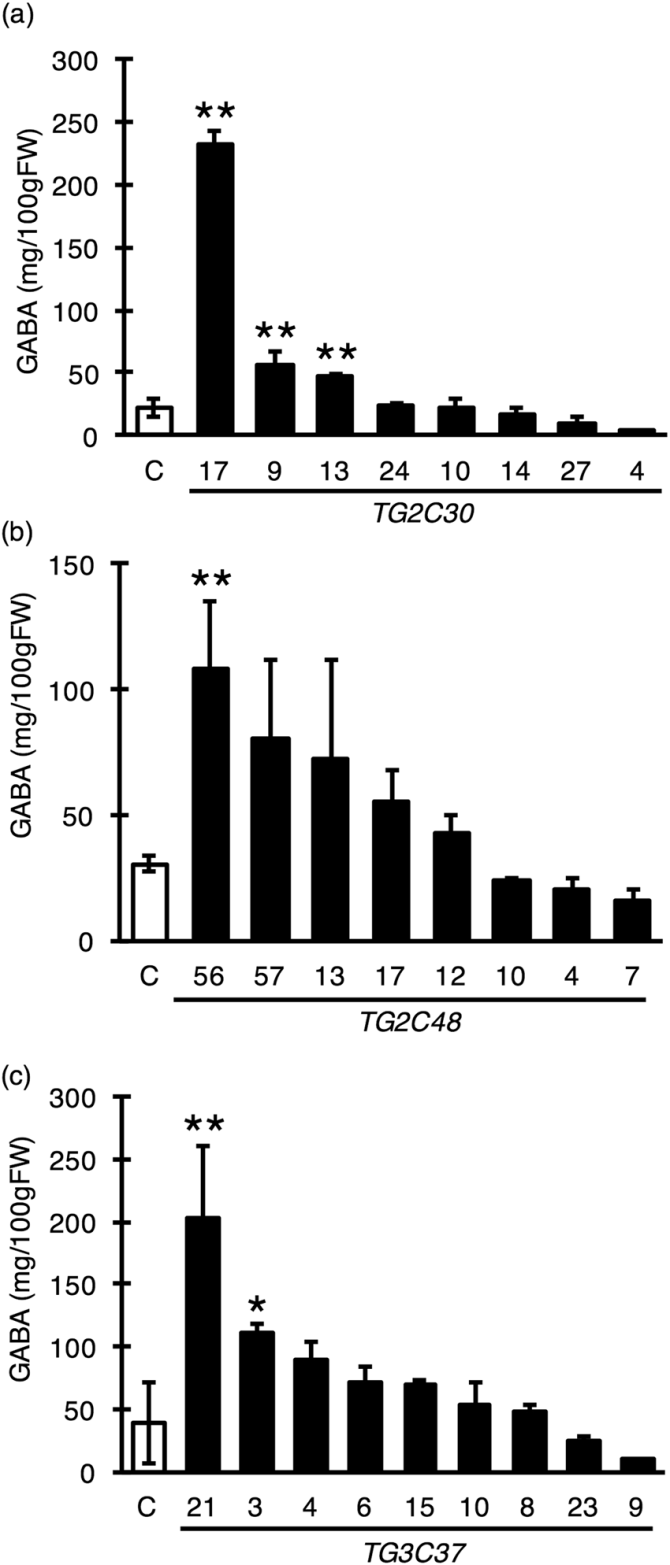
GABA content of T_0_ red-stage tomato fruits. GABA content was measured by the GABase enzymatic method using fruits at the Breaker-stage +10 days in the T_0_ generation. Mutations were detected at the target site of CRISPR/Cas9 in all lines. Bars indicate standard deviation (n = 3) and asterisks statistical differences according to the Tukey-Kramer’s test between mutated and control lines (*P < 0.05 and **P < 0.01). ‘C’ indicates the control plant with wild type (WT) genotype. (**a**,**b** and **c**) show GABA content in TG2C30, TG2C48, and TG3C37 mutated lines, respectively.

We examined the effect of the SlGAD mutations on GABA accumulation in red-stage tomato fruits (10 days after the Breaker stage). The GABA accumulation was measured by the GABase assay method, which is a simple test to detect GABA (Fig. 2). In TG2C30, three of the eight mutant lines (#17, #9, #13) showed 2 to 10 times more GABA than the WT, and the highest-accumulation line presented 232.9 ± 7.4 mg/100 g fresh weight (FW) (n = 3, Fig. 2a). Five lines of TG2C48 (#56, #57, #13, #17, #12) showed 1.5 to 3.6 more GABA that the WT, with the highest value corresponding to 108.5 ± 26.57 mg/100 g FW (Fig. 2b). Seven lines of TG3C37 (#21, #3, #4, #6, #15, #10, #8) had higher GABA accumulation than the WT, reaching 203.3 ± 31.7 mg/100 g FW (Fig. 2c). The other lines in TG2C30 (#24, #10, #14, #27, #4), TG2C48 (#10, #4, #7), and TG3C37 (#23, #9) showed similar GABA levels to WT.

### Relationship between C-terminal mutation types and GABA accumulation in T_0_

The mutation types of the C-terminal were reflected in the GABA accumulation levels of the T_0_ generation (Table 2, Supplementary Fig. 2). Type 1 and Type 3 mutants showed higher GABA accumulation whereas Type 2 and Type 4 mutants showed similar levels to the WT. These results indicated that a truncated C-terminal region immediately upstream of the autoinhibitory domain effectively increased GABA accumulation.

### Genotype and zygosity in T_1_ plants

The zygosity and genotype of target sites in the T_1_ generation were examined as previously described^34^. Since this method used PCR product of target site and sequencing directly, homozygotic and WT genotypes was clearly identified whereas bi-allelic, heterozygous, and chimeric lines were not (identified as ‘h’ in Table 1). Six TG2C30 (#9, #13, #14, #17, #24, and #27), four TG2C48 (#13, #10, #56, and #57), and four TG3C37 (#3, #4, #6, and #21) lines that exhibited high GABA levels in the T_0_ generation and/or had enough seeds were used for experiments. Among these lines, 44.0% (107/243) were homozygotes, 15.2% (37/243) were WT, and 40.7% (99/243) were ‘h’. The 107 homozygous T_1_ plants had three genotypes: deletion ranging from a single to more than 200 nucleotides (66.4%, 71/107), single-nucleotide insertion (30.8%, 33/107), and simultaneous insertion and deletion (2.8%, 3/107).

### Structure of GADs C-terminal in the homozygous mutant line of T_1_

Amino acid sequences of mutation lines in T_1_ homozygous are summarized in Table 3. Because all of these plants were homozygous, the structure of their C-terminal was classified into Type 1 or Type 2; this was further divided in two groups: C-terminal Type 2a had a conserved autoinhibitory domain and Type 2b was a defective autoinhibitory domain in C-terminal, which had almost the same size as that of WT (Supplementary Fig. 3

**Table 3:** Amino acid sequence of Target site (T1 generation).

Line	Genotype	Amino sequence		
GAD2	WT	**DIV-10AA-RVKAKLAVAEEAAAANGIDSVAHH***K*TDREWELQVTEAWKKFVADKKKNKTMGVC*	Type of C-terminal	GABA
*TG2C30*				
#17-2	d50	**DIV-10AA-RVKAKLAVAEEAAAANGIDSV**EEVCC*	1	Higher
#17-4	d50	**DIV-10AA-RVKAKLAVAEEAAAANGIDSV**EEVCC*		
#17-1	d3	**DIV-10AA-RVKAKLAVAEEAAAANGIDSVAHH**-TDREWELQVTEAWKKFVADKKKNKTMGVC*	2a	Same
#17-3	d3	**DIV-10AA-RVKAKLAVAEEAAAANGIDSVAHH**-TDREWELQVTEAWKKFVADKKKNKTMGVC*		
#9-3	WT	**DIV-10AA-RVKAKLAVAEEAAAANGIDSVAHH***K*TDREWELQVTEAWKKFVADKKKNKTMGVC*	2a	Same
#9-5	WT	**DIV-10AA-RVKAKLAVAEEAAAANGIDSVAHH***K*TDREWELQVTEAWKKFVADKKKNKTMGVC*		
#9-7	WT	**DIV-10AA-RVKAKLAVAEEAAAANGIDSVAHH***K*TDREWELQVTEAWKKFVADKKKNKTMGVC*		
GAD2	WT	**DIV-10AA-RVKAKL***A*VAEEAAAANGIDSVAHHKTDREWELQVTEAWKKFVADKKKNKTMGVC*	Type of C-terminal	GABA
*TG2C48*				
#13-6	i1	**DIV-10AA-RVKAKL**GRGRGGGRGERYRQRGTS*	1	Higher
#56-6	i1	**DIV-10AA-RVKAKL**GRGRGGGRGERYRQRGTS*	1	Higher
#56-18	i1	**DIV-10AA-RVKAKL**GRGRGGGRGERYRQRGTS*		
#56-19	i1	**DIV-10AA-RVKAKL**GRGRGGGRGERYRQRGTS*		
#56-24	i1	**DIV-10AA-RVKAKL**GRGRGGGRGERYRQRGTS*		
#13-1	WT	**DIV-10AA-RVKAKL***A*VAEEAAAANGIDSVAHHKTDREWELQVTEAWKKFVADKKKNKTMGVC*	2a	Same
#56-1	WT	**DIV-10AA-RVKAKL***A*VAEEAAAANGIDSVAHHKTDREWELQVTEAWKKFVADKKKNKTMGVC*	2a	Same
#56-13	WT	**DIV-10AA-RVKAKL***A*VAEEAAAANGIDSVAHHKTDREWELQVTEAWKKFVADKKKNKTMGVC*		
#56-17	WT	**DIV-10AA-RVKAKL***A*VAEEAAAANGIDSVAHHKTDREWELQVTEAWKKFVADKKKNKTMGVC*		
#56-12	d27	**DIV-10AA**----------EEAAAANGIDSVAHHKTDREWELQVTEAWKKFVADKKKNKTMGVC*	2a	Same
#13-4	d7	**DIV-10AA-RVKA**PWPRRRPRRTVSTAWHIIKRIENGSYRLLKHGRSLLLIKRRIRLWEFV*	2b	Lower
#13-16	d7	**DIV-10AA-RVKA**PWPRRRPRRTVSTAWHIIKRIENGSYRLLKHGRSLLLIKRRIRLWEFV*		
#13-3	d1	**DIV-10AA-RVKAKL**PWPRRRPRRTVSTAWHIIKRIENGSYRLLKHGRSLLLIKRRIRLWEFV*	2b	Lower
#13-9	d1	**DIV-10AA-RVKAKL**PWPRRRPRRTVSTAWHIIKRIENGSYRLLKHGRSLLLIKRRIRLWEFV*		
#13-14	d1	**DIV-10AA-RVKAKL**PWPRRRPRRTVSTAWHIIKRIENGSYRLLKHGRSLLLIKRRIRLWEFV*		
#13-17	d1	**DIV-10AA-RVKAKL**PWPRRRPRRTVSTAWHIIKRIENGSYRLLKHGRSLLLIKRRIRLWEFV*		
#13-18	d1	**DIV-10AA-RVKAKL**PWPRRRPRRTVSTAWHIIKRIENGSYRLLKHGRSLLLIKRRIRLWEFV*		
GAD3	WT	**TLADRLVSDIVKVLHELPNAKK***V*EDNLMINNEKKTEIEVQRAIAEFWKKYVLARKASIC*	Type of C-terminal	GABA
*TG3C37*				
#21-1	i1	**TLADRLVSDIVKVLHELPNAKK***V*GG*	1	Higher
#21-5	i1	**TLADRLVSDIVKVLHELPNAKK***V*GG*		
#21-7	i1	**TLADRLVSDIVKVLHELPNAKK***V*GG*		
#21-16	i1	**TLADRLVSDIVKVLHELPNAKK***V*GG*		
#21-18	d53	TLADRLVSDIVKVLHELPN*	1	Higher
#21-19	d53	TLADRLVSDIVKVLHELPN*		
#21-26	d53	TLADRLVSDIVKVLHELPN*		
#3-1	d6i1	**TLADRLVSDIVKVLHELPNA**IGG*	1	Higher
#3-6	d6i1	**TLADRLVSDIVKVLHELPNA**IGG*		
#3-8	d6i1	**TLADRLVSDIVKVLHELPNA**IGG*		
#3-4	d4	**TLADRLVSDIVKVLHELPNA**KWRII*	1	Higher
#3-10	d4	**TLADRLVSDIVKVLHELPNA**KWRII*		
#3-12	d4	**TLADRLVSDIVKVLHELPNA**KWRII*		
#3-13	d4	**TLADRLVSDIVKVLHELPNA**KWRII*		

Genotype of all plants in this table were homozygous. WT, wild-type sequence with no mutation detected. “d” and “i” means deletion and insertion, respectively. “*” was stop codon. The same sequence as Wild Type is expressed in **Bold**. The changed sequence by the mutation via CRISPR/Cas9 was in Regular. **Bold with underline** is the putative auto inhibitory domain and CaMd domain. The target site is in *Italic*. “a” and “b” in Superscript mean different one base-pair deletion. “Higher” meant higher GABA content comparing with WT. “Lower” shows lower GABA content than WT. “Same level” indicated almost the same contain level as WT. GABA content was measured by HPLC, using leaf (TG3C30 and TG2C48) and fruits at the Br +10 stage (red) (TG3C37).

). The most frequent mutation was Type 1 (21/38 plants), the second was Type 2a (10/38 plants), and the rarest was Type 2b (7/38 plants). Type frequency differed according to the target site. In TG2C30, the most frequent mutation was Type 2a (5/7 plants), in TG2 C48 it was Type 2b (7/17 plants), and in TG3C37 it was Type 1 (all plants).

### Plant size, flowering, and fruit yield in the T_1_ generation

**Figure 3:**
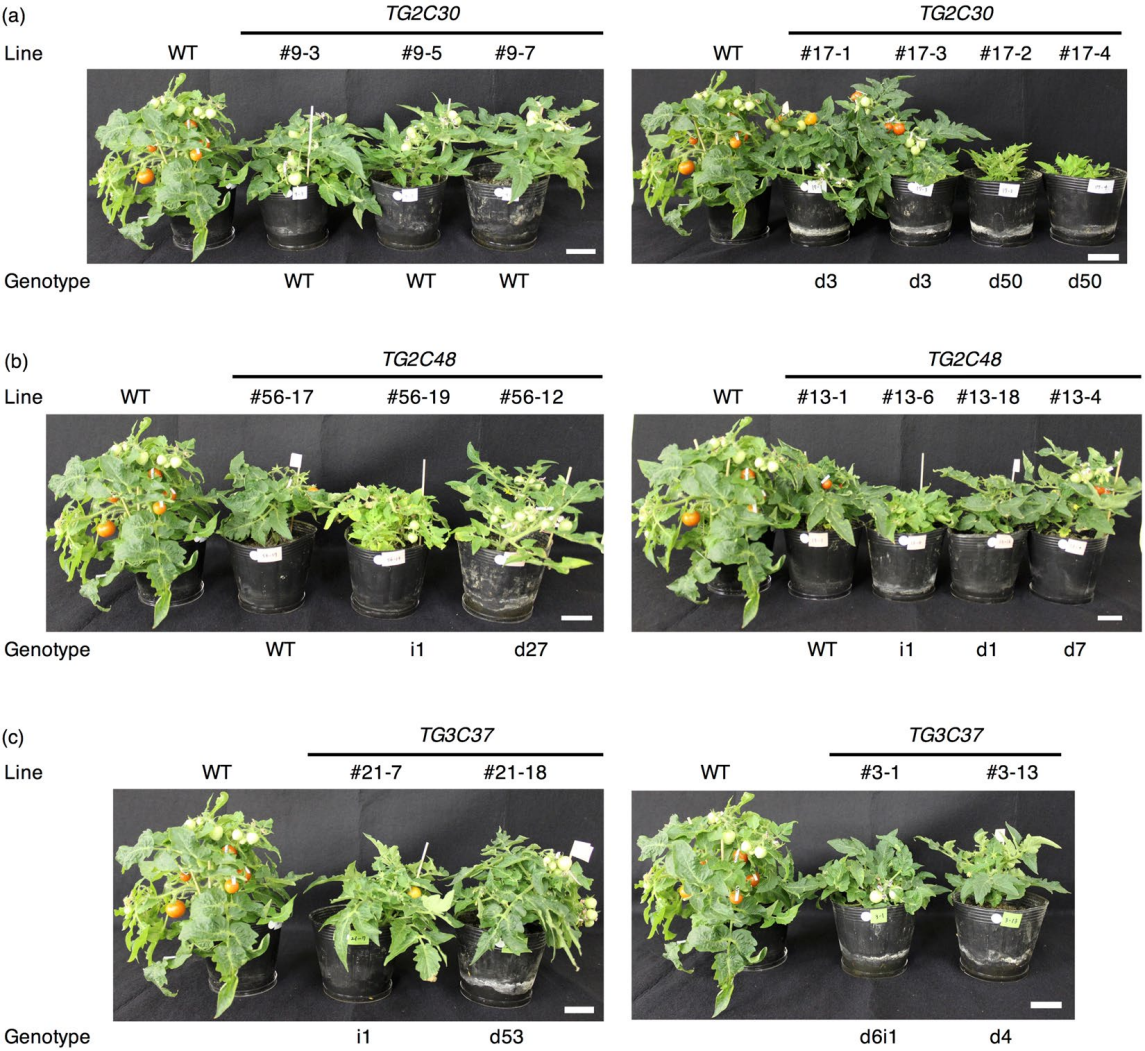
Plant size in 25-week-old plants in the T_1_ generation. All plants were homozygous. Genotype ‘WT’ was identical to that of wild type, as no mutation was detected. ‘d’ and ‘i’ indicate deletion and insertion, respectively. Line ‘WT’ indicates a wild type plant. White bars correspond to 5 cm.

The phenotype of 38 homozygous plants from six lines whose red-stage fruit showed high GABA content in T_0_ (Fig. 2) were analyzed in the T_1_ generation. This included 22 plants that were null CRISPR/Cas9 segregates. All TG2C30, TG2C48, and TG3C37 plants except for TG2C30 (genotype d50; #17-2, #17-4) and TG2C48 (genotype i1; #56-6, #56-18, #56-19, #56-24, #13-6) were slightly smaller than WT (Fig. 3 and Table 4). A similar trend was observed in the TG2C30 lines (#9-3, #9-5, #9-7) with the WT genotype. All of these lines were produced by regeneration, which could affect plant size in the T_1_ generation. TG2C30 (genotype d50; #17-2, #17-4) and TG2C48 (genotype i1; #56-6, #56-18, #56-19, #56-24, #13-6) plants were remarkably small (Table 4). Flowering was delayed in TG2C48 (#56-19), and there were no flower buds produced by TG2C30 (genotype d50; #17-2 and #17-4) and TG2C48 (genotype i1; #56-6, #56-18, #56-19, #56-24, and #13-6). Differences in the flowering were not observed between the TG2C30 (genotype d3; #17-1, #17-3), TG2C48 (genotype d1; #13-3, #13-4, #13-14, #13-17 and genotype d7; #13-9, #13-16, #13-18), all lines of TG3C37 and WT lines (Table 4). Red-stage (Breaker +10 days) fruit number per plant in the TG2C30 (genotype d3; #17-1, #17-3), TG2C48 (genotype d1; #13-3, #13-9, #13-14, #13-17, #13-18), and all lines of TG3C37 was almost the same as WT (Table 4), and red-stage fruit size was similar to or slightly larger than WT (Supplemental Fig. 4). Thus, fruit yield was not significantly affected in these lines. On the other hand, fruit number was low in TG2C30 (genotype d50; #17-2, #17-4) and TG2C48 (Genotype i1; #56-6, #56-18, #56-19, #56-24, #13-6) relative to WT.

**Table 4:** Characterization of T_1_ generation.

	Line	Genotype	Plant Number	Fruit Number	Days until flowering (Days)	Height (mm)	GABA accumulation (mg/gFW)	
							Leaf	Fruit
WT			5	51<	49.7 ± 2.0	99.2 ± 6.6	4.9 ± 0.92 (n = 15)	10.04 ± 2.0 (n = 15)
*TG2C30*	9	WT	13	112<	64.5 ± 6.3	88.1 ± 7.2	2.79 ± 0.58 (n = 9)	n.t.
	17	d3	2	21<	n.t.	n.t.	2.9 ± 1.86 (n = 6)	n.t.
		d50	2	0	n.t.	n.t.	98.2 ± 24.31** (n = 6)	n.t.
*TG2C48*	56	WT	6	57<	57.7 ± 4.6	92.8 ± 5.3	3.8 ± 1.43 (n = 9)	n.t.
		i1	4	4	85.0 ± 7.4**	63.6 ± 6.8*	130.62 ± 41.03** (n = 12)	n.t.
		d27	1	13<	n.t.	n.t.	3.92 ± 1.07 (n = 3)	n.t.
	13	WT	1	9<	n.t.	n.t.	2.05 ± 0.86 (n = 3)	n.t.
		d1	5	60<	52.6 ± 3.4	77.4 ± 2.3	0.32 ± 0.11^†^ (n = 15)	n.t.
		d7	2	20<	n.t.	n.t.	0.31 ± 0.12^†^ (n = 6)	n.t.
		i1	1	0	n.t.	n.t.	139.55 ± 44.65** (n = 3)	n.t.
*TG3C37*	21	i1	4	37<	74.5 ± 3.5	108.9 ± 7.1	5.18 ± 2.26 (n = 12)	78.0 ± 11.31** (n = 12)
		d53	3	28<	57.3 ± 10.0	96.0 ± 6.2	5.27 ± 1.00 (n = 9)	88.72 ± 15.9** (n = 9)
	3	d4	4	38<	50.0 ± 5.4	77.2 ± 4.1	3.77 ± 1.77 (n = 12)	78.75 ± 20.75** (n = 12)
		d6i1	3	26<	63.3 ± 10.4	71.8 ± 7.3	3.17 ± 0.75 (n = 9)	94.94 ± 32.44 (n = 9)

Genotype of all plants in this table were homozygous. In “Genotype”, WT was same as wild-type sequence with no mutation detected. “d” and “i” means deletion and insertion, respectively. In column of “Days until flowering” and “Height”, data represent means ± SD, the repetition was same as plant number. GABA content data showed means ± SD (repetition). Asterisk and dagger indicated statistical differences in *Tukey-Kramer’s* test and *Student’s t-test* (*P > 0.05, **P > 0.01, ^†^*P* > 0.01), respectively. “n.t.” was non-tested.

### GABA accumulation in leaves of T_1_ plants

**Figure 4:**
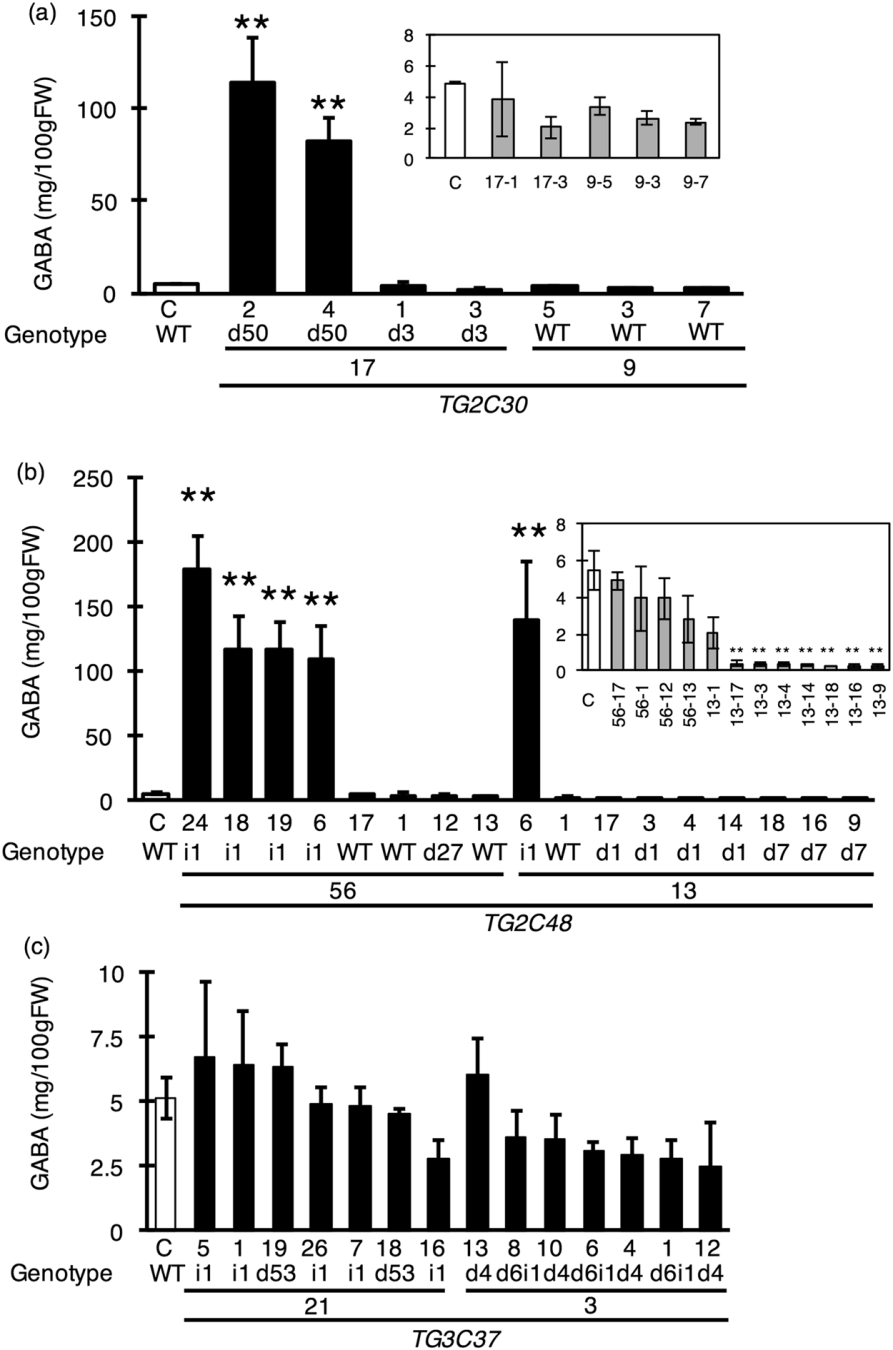
Leaf GABA content in the T_1_ generation. GABA content of leaves was measured by HPLC. All lines were homozygous. Bars indicate standard deviation (n = 3) and asterisks indicate statistical differences in relation to control according to the Tukey-Kramer’s test (*P < 0.05 and **P < 0.01). ‘C’ is the control plant with WT genotype. (**a**,**b** and **c**) show GABA content in TG2C30, TG2C48, and TG3C37 mutated lines, respectively.

Plant size was markedly reduced in TG2 C30 (genotype d50; #17-2 and #17-4) and TG2C48 (genotype i1; #56-6, #56-18, #56-19, #56-24, and #13-6) (Fig. 3 and Table 4). Previous studies in *Arabidopsis thaliana* reported that GABA accumulation affected cell elongation and decreased plant size^35^. Accordingly, we speculated that the observed reduction in plant size was caused by high GABA levels. To verify this possibility, we measured the GABA content in leaves by high-performance liquid chromatography (HPLC). TG2C30 (genotype d50; #17-2, #17-4) and TG2C48 (genotype i1; #56-6, #56-18, #56-19, #56-24, #13-6) had 16.5- to 36-fold higher GABA levels than WT plants, with the highest value corresponding to 179.01 ± 25.16 mg/100 g FW (Fig. 4b). On the other hand, TG2C48 (genotype d1; #13-3, #13-4, #13-14, #13-17 and genotype d7; #13-9, #13-16, #13-18) had levels that were lower than that of the WT. GABA content in the other lines TG2C30 (genotype d3; #17-1, #17-3), TG2C48 (genotype d1; #13-3, #13-9, #13-14, #13-17, #13-18), and all lines of TG3C37 were comparable to that in WT (Fig. 4). Only lines that had a smaller plant size had high GABA levels in the leaves; this was associated with decreased flowering and fruit yield.

### Correlation between GAD C-terminal structure and GABA accumulation

The structure of the C-terminal was correlated with GABA accumulation (Table 3, Supplementary Fig. 3). Type 1 mutants with the truncated C-terminal had higher GABA accumulation than the WT, whereas Type 2a mutants, with the conserved autoinhibitory domain, had a similar GABA accumulation to that of the WT; Type 2b showed a reduced GABA accumulation. These results indicated that the truncation of the autoinhibitory domain in the C-terminal region (Type 1) increased GABA accumulation, and that in Type 2b the presence of additional meaningless amino acids strongly reduced GABA accumulation.

### GABA accumulation in red-stage fruits of T_1_ plants

**Figure 5:**
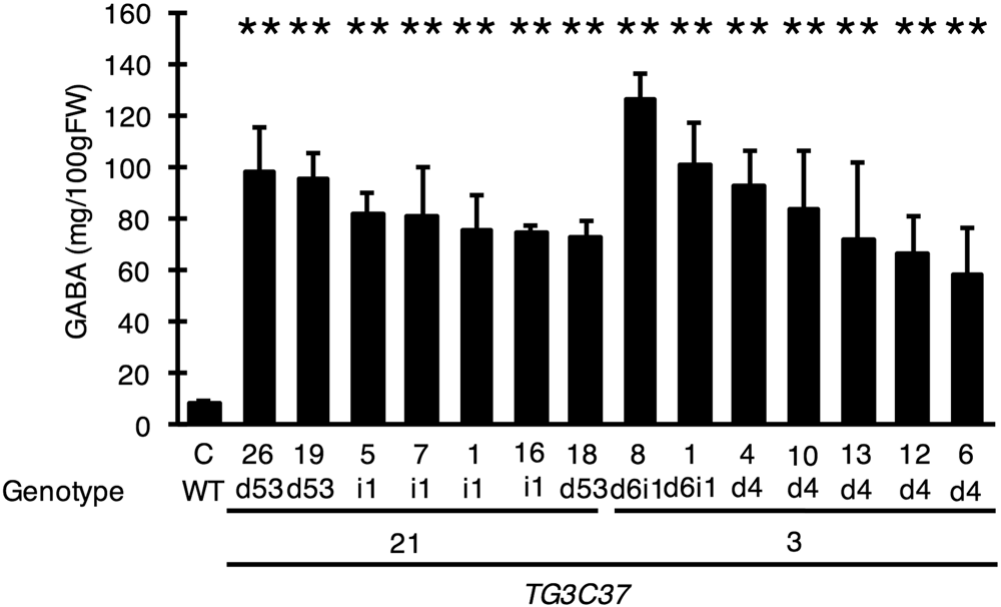
Red-stage fruit GABA content in the T_1_ generation. GABA content of red-stage fruits (Breaker-stage +10 days) was measured by HPLC. All lines were homozygous for TG3C37. Bars indicate standard deviation (n = 3) and asterisks indicate statistical differences in relation to control according to the Tukey-Kramer’s test (*P < 0.05 and **P < 0.01). ‘C’ is the control plant with WT genotype.

TG3C37 included only Type 1 mutants with potentially higher GABA content. We examined the effect of this mutation in red-stage (Breaker +10 days) fruit suitable for harvest by HPLC analysis of GABA levels. All TG3C37 lines had greater-than-WT GABA accumulation. TG3C37 #3 produced seven to 15 times more GABA than WT plants, with a maximum value of 125.73 ± 6.17 mg/100 g FW (Fig. 5). GABA accumulation in TG3C37 #21 was 8.75 to 11.6 times higher than in WT, with the highest value being 97.84 ± 17.46 mg/100 g FW (Fig. 5). All of the lines with high GABA content in the fruit had a stop codon immediately upstream of the autoinhibitory domain and close to C37 (ΔC34, ΔC36, and ΔC40). Taken together with the results of the *in vitro* enzyme assay of SlGAD3ΔC37 (Fig. 1c), we conclude that C-terminal truncation of SlGAD3 increases enzymatic activity, leading to GABA accumulation in the tomato fruit.

## Discussion

Our results found that SlGAD2 and SlGAD3 lacking the C-terminal had an increased activity (Fig. 1b,c). Thus, the C-terminal of SlGAD2 also includes the autoinhibitory domain, and tomato has at least two kinds of active GADs. The activity of SlGAD2 is almost 10 times higher than that of SlGAD3 at pH 5.8 (Fig. 1b,c). The alignment of SlGAD2 and SlGAD3 amino acid sequences (Supplementary Fig. 5) showed these are well conserved, except for the C-terminal region, as SlGAD2 presented additional sequence immediately upstream of autoinhibitory domain compared to SlGAD3. In apple (*Malus domestica*), MdGAD1 showed higher activity than MdGAD2^36^ and MdGAD1 also had additional sequence immediately upstream of the autoinhibitory domain (Supplementary Fig. 6). These results suggest that this additional sequence immediately upstream of the autoinhibitory domain might affect the strength of GAD activity.

The correlation between the C-terminal mutation Types and GABA accumulation was observed in the T_0_ generation (Table 2, Supplementary Fig. 2). It was found through the analysis of the Types of C-terminal mutation and GABA accumulation that at least one truncated C-terminal effectively increased GABA accumulation. This means that heterozygous GADs with truncated C-terminal might increase GABA accumulation in the T_0_ generation. In tomato breeding, F_1_ hybrids are frequently used. The analysis of the C-terminal in the T_0_ generation and GABA accumulation suggested that the F_1_ hybrid lines would exhibit high GABA content. Thus, either parental line with a truncated GAD C-terminal introduced by CRISPR/Cas9 may be effectively used for high-GABA-content tomato breeding.

The result of our experiment, in T_1_ generation, clearly showed that completely removal of the C-terminal region (Type 1) increased the GABA accumulation and defective C-terminal (Type 2b) caused the reduction (Table 3, Supplementary Fig. 4). The C-terminal has functions for autoinhibitory domain activated via Ca^2+^/CaM^20, 21, 37^. Thus, our results suggest that although the removal of the C-terminal increased GAD activity^21^ (Fig. 1a), deficiencies in this domain would lead to the complete loss of GAD activity and reduce GABA accumulation in tomato. In cell environmental conditions, i.e., at pH 7.0, the C-terminal of GAD works as ‘cover’ for the active site of the enzyme, but under GABA-required conditions, such as response to biotic or abiotic stresses and growth and development, the autoinhibitory domain is released via the active site is uncover via Ca^2+^/CaM binding^20, 37^. Our study suggests that lines with the defective C-terminal (Type 2b) might have lost the function of Ca^2+^/CaM binding, keeping the active site ‘covered’, resulting in low GABA accumulation.

SlGAD2 with lacking the C terminus (Type 1) suppressed plant growth, flowering, and fruit yield (Table 4), which was associated with abnormally high GABA levels in the leaves. In contrast, SlGAD3 with truncation of C terminal (Type 1) showed leaf GABA accumulation that was comparable to WT. The effect of this mutation on plant size was less than that of SlGAD2 lacking the C terminus. Moreover, this mutation increased GABA accumulation in fruits without decreasing flowering and fruit yield (Fig. 3 and Table 4). We therefore conclude that SlGAD3 is a more suitable CRISPR/Cas9 target site than SlGAD2 for increasing GABA levels in tomato fruits. The differential effects of mutations in the two genes are likely due to their distinct expression patterns: *SlGAD2* and *SlGAD3* were most highly expressed in leaves and in fruits, respectively (Supplemental Fig. 7).

Remarkably smaller plant size in TG2C30 (genotype d50) and TG2C48 (genotype i1) with (Type 1) was caused by increased GABA accumulation in leaves (Fig. 4a,b). Altered concentrations of GABA and related metabolites *in vivo* can lead to severe developmental defects such as short stems caused by the failure of parenchyma cell elongation^21–23^. GAD is the entry enzyme of the GABA shunt that catalyses the decarboxylation of glutamic acid (Glu) to GABA^38^; increasing this activity can lead to a major change in carbon-nitrogen (C-N) balance that favours N^23^. Moreover, high levels of GABA in *A. thaliana* decreased the expression of genes encoding secreted and cell wall-related proteins^35^. SlGAD2 activation via removal of the autoinhibitory domain may thus enhance Glu-to-GABA decarboxylation and alter C-N balance, and high GABA content in leaves could inhibit the expressions of genes related to cell elongation, thereby decreasing plant size.

The CRISPR/Cas9 system is more effective than the TILLING platform for introducing mutations at specific sites, for instance in the 30–50 amino acids of the SlGAD C terminus. In tomato, 0.5% and 1.0% EMS populations harbour one mutation per 1,710 and 737 kb, respectively. The overall mutation frequency (one mutation per 1,237 kb)^24^ affects an average of three alleles per screened kilobase; thus, the TILLING platform is more useful for screening relatively wide regions within the genome. In a previous study using 4,588 lines of EMS mutant alleles, we isolated a GAD mutant with the TILLING platform (Ezura *et al*., unpublished data). Although lines with four *SlGAD2* and two *SlGAD3* mutations were identified, each mutation was located around the N terminus of SlGAD2 or SlGAD3. *SlGAD2* and *SlGAD3* genes are 6.7 kb and 2.0 kb, respectively; given the average mutation frequency, we likely isolated the maximum number of mutant lines for each gene, and it would have been difficult to isolate SlGAD2ΔC and SlGAD3ΔC in these EMS populations using the TILLING platform. The present study showed that screening 14 to 35 transgenic lines with the CRISPR/Cas9 vector was sufficient to introduce mutations at the C terminus of SlGADs, demonstrating its superiority for introducing site-specific or small-area mutations.

Transgenic strategies have been employed to increase GABA accumulation in tomato fruits^18, 19^. Although overexpressing full-length SlGAD3 containing 155 mg/100 g FW GABA did not affect fruit ripening, overexpressing SlGAD3 lacking the C terminus (268 mg/100 g FW) inhibited the development of fruits with a red-ripe coloration from those with an orange colour^19^. From these previous results, we estimated that the maximum concentration of GABA in a normal ripe fruit is 155 mg/100 g FW. The targeted mutagenesis through the CRISPR/Cas9 system deleted the autoinhibitory domain of C-terminal in SlGAD3 and increased the GABA accumulation up to 125 mg/100 g FW (15 times higher than WT) in fruit with normal-red ripening. This result means that the targeted mutagenesis is useful strategy to increase GABA accumulation as well as transgenic. Regarding breeding, the application of mutagenesis would be more acceptable than transgenesis. Because after the segregation of CRISPR/Cas9 vector, it would be un-distinguished the product via conventional mutation technique and targeted mutagenesis. Therefore, inducing a mutation in the C-terminal of SlGAD3 via CRISPR/Cas9 seems to be a more useful method than the transgenic strategy with respect to both fruit phenotype and public acceptance in near feature.

We succeeded in producing a high-GABA-accumulation tomato line (up to 125 mg/100 g FW in red ripe tomato fruits) using the targeted mutagenesis through the CRISPR/Cas9 system. The daily intake of 10–20 mg of GABA effectively reduces blood pressure in adults with mild hypertension^11, 39, 40^. This amount of GABA is equivalent to the level present in 10–20 g FW of C-terminal-removed tomato, which corresponds to 3–6 ‘Micro-Tom’ fruits and to one-tenth of a large tomato fruit. Therefore, the removal of SlGAD3 C-terminal by targeted mutagenesis seems to be sufficient to increase GABA accumulation and for promoting health.

The removal of the C-terminal in GADs increased their activity in many plant species, such as petunia, rice, and tomato^19, 22, 41^, and the overexpression of truncated C-terminal in GADs remarkably increased GABA accumulation in tobacco, rice, *A. thaliana*, and tomato^19, 21–23^. In apple, the activity of MdGAD1 and MdGAD2 is regulated by their C-terminal with a Ca^2+^/CaM binding site^36^. In the present study, the mutation introduced into the C-terminal of SlGADs, which regulates their enzymatic activity^42, 43^, using the targeted mutagenesis, CRISPR/Cas9 system, produced high-GABA-accumulation tomato line. Thus, removing the C-terminal of GAD effectively increases GABA accumulation in a wide variety of crops. Therefore, our study suggests that the utilization of targeted mutagenesis targeting the C-terminal of GADs would be effective to breed high-GABA-accumulation varieties in wide range of crops. This strategy might contribute to recover from diseases associated with lifestyle, such as high blood pressure, via dietary products.

## Methods

### Plant materials and growth conditions

Tomato (*Solanum lycopersicum* L.) ‘Micro-Tom’ seeds were provided by the National Bioresource Project^15^. Seeds of WT and T_1_ generation were germinated on moistened filter paper, transplanted into soil, and grown in a culture room at 25 °C, under fluorescent light (60 µmol/m^2^/s) in a 16:8 h light:dark photoperiod. A standard nutrient solution (Otsuka A; Otsuka Chemical Co., Ltd, Osaka, Japan) was supplied. Plant height was measured on the first flowering day. Leaves were sampled during vegetative growth. Fruits were harvested 10 days after the Breaker-stage.

### Vector construction and tomato transformation

The vectors for the CRISPR/Cas9 system (pZD_AtU6_Hpger_Cas9_NPTII and pDeCas9_Kan)^44, 45^ were kindly provided by Prof. Holger Puchta (Botanical Institute II, Karlsruhe Institute of Technology, Karlsruhe, Germany) and Prof. Seiichi Toki [National Agriculture and Food Research Organization (NARO), Japan], respectively. The pZD_AtU6_Holger_Cas9_NPTII vector was used to introduce the mutation in the C-terminal of SlGAD2 at nucleotide 90 (corresponding to 30 amino acids), whereas pDeCas9_Kan was used to develop SlGAD2ΔC48 and SlGAD3ΔC37. The modification of these plasmids was performed following methods presented in previous studies^30, 44, 45^. Constructed vectors were introduced into *A. tumefacien*s GV2260^46^ via electroporation. Using a highly efficient protocol^33^, CRISPR/Cas9 expression was introduced into the tomato genome. Only diploid plants were screened and used in the following experiments.

### Determination of GAD activity in *in vivo* assays

SlGAD2ΔC30, SlGAD2ΔC48, and SlGAD3ΔC37 were cloned into pET-32a vectors (Promega), which were transformed into the *E. coli* strain BL21(DE3) pLysS. Expression was induced by 1 mM isopropyl-β-D(-)-thiogalactopyranoside. The recombinant protein, with a histidine (His) tag, was purified by HisTrap™FF (GE Healthcare, Buckinghamshire, UK). The activity of GAD was measured according to previous study^22^, by directly measuring GABA production after GAD reactions. The production of GABA was measured by the GABase method, as described below.

### Measuring GABA content by the GABase method

Leaves and Breaker stage +10 days fruits were harvested and immediately frozen in liquid nitrogen. Frozen leaves and fruits were ground in liquid nitrogen, and free amino acids were extracted from 50 mg of frozen powdered samples with 8% (w/v) trichloroacetic acid. Samples were centrifuged at 10,000 × *g* for 20 min at 4 °C, and the supernatant was transferred to a new tube: 400 µl of pure diethyl ether were added, and samples were vortexed for 10 min. The mixture was centrifuged at 10,000 × *g* for 10 min at 4 °C and, after removing the supernatant, diethyl ether was added and the sample was vortexed for another 10 min. A second centrifugation at 10,000 × *g* for 10 min at 4 °C was performed and, after removing the supernatant, the samples were left under a draft for 1 h to completely remove diethylene. The GABA content was measured using the GABase method described by Jakoby^47^, with a slight modification according to Akihiro *et al*.^17^, or using the HPLC method, as described by Koike *et al*.^14^, using completely dried samples dissolved in 0.01N HCl.

### Statistical analysis

Mean values were compared using the Tukey-Kramer’s test. Data are expressed as means ± S.D. The Tukey-Kramer’s test was used to detect significant differences between the control. The level of statistical significance was set at*P < 0.05 and **P < 0.01.

## Electronic supplementary material

Supplementary Information 
