# Author Correction: Efficient increase of ɣ-aminobutyric acid (GABA) content in tomato fruits by targeted mutagenesis

**DOI:** 10.1038/s41598-019-55119-5

**Published:** 2019-12-19

**Authors:** Satoko Nonaka, Chikako Arai, Mariko Takayama, Chiaki Matsukura, Hiroshi Ezura

**Affiliations:** 1 Faculty of Life and Environmental Sciences, University of Tsukuba, 1-1-1 Tennodai, Tsukuba, Ibaraki 305-8572 Japan; 2 Gene Research Center, University of Tsukuba, 1-1-1 Tennodai, Tsukuba, Ibaraki 305-8572 Japan; 3 Graduate School of Life and Environmental Sciences, University of Tsukuba, 1-1-1 Tennodai, Tsukuba, Ibaraki 305-8572 Japan

Correction to: *Scientific Reports* 10.1038/s41598-017-06400-y, published online 01 August 2017

This Article contains errors in Figure 1B and 1C where the units are incorrect. The correct Figure 1 appears below.

**Figure 1:**
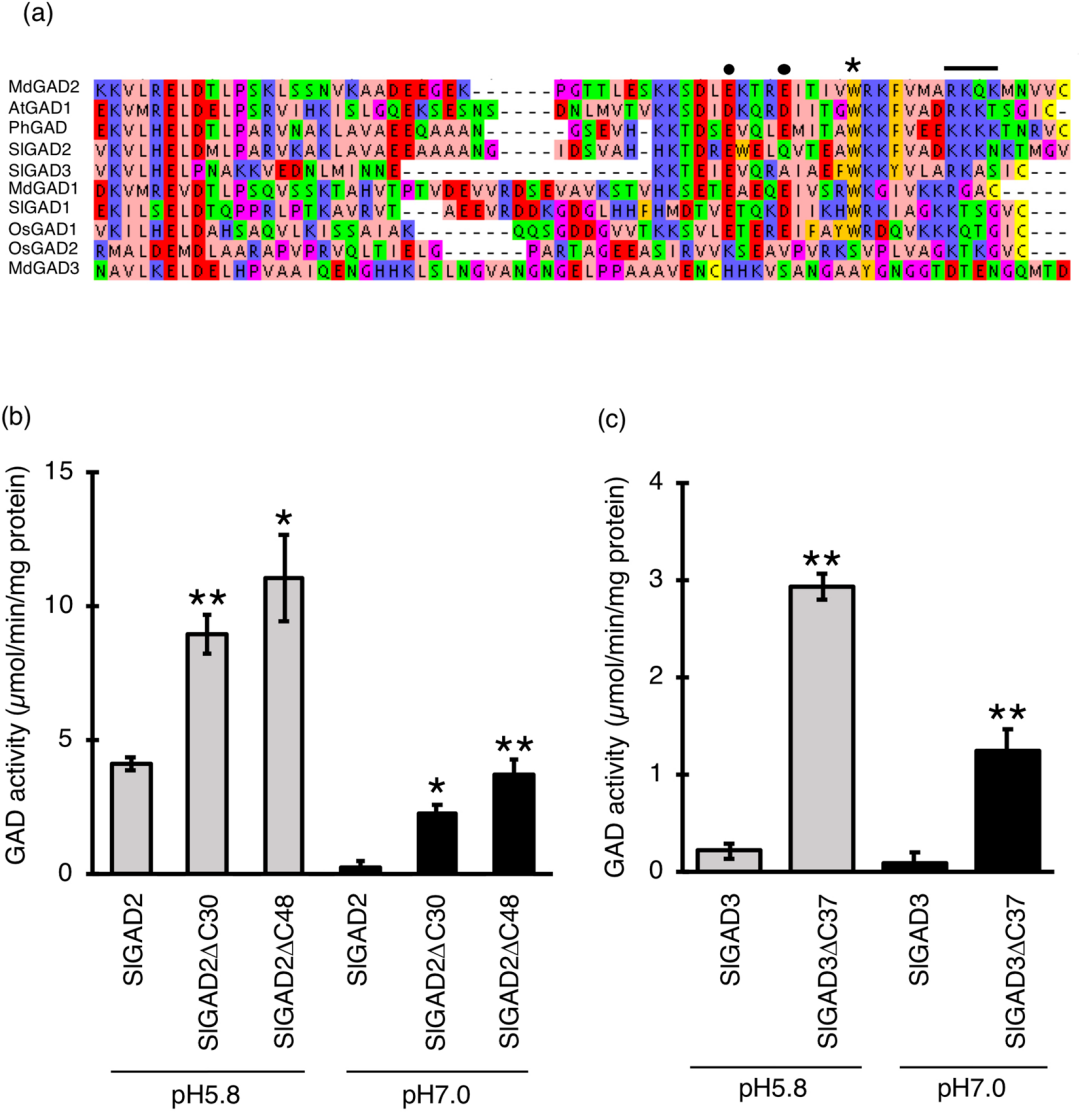
.

In addition, the legend for Figure 1 is incorrect. The Figure legend,

“Crude extracts from Escherichia were used in GAD enzymatic assays and Ca2+ was not included in reaction buffers.”

should read:

“Recombinant GAD proteins were expressed in Escherichia coli, purified from the bacterial crude extracts and used in the GAD enzymatic assays.”

