# Supplementary material for: Efficient increase of ɣ-aminobutyric acid (GABA) content in tomato fruits by targeted mutagenesis
Source: Sci Rep. 2017 Aug 1;7:7057. doi: 10.1038/s41598-017-06400-y (PMC5539196; doi:10.1038/s41598-017-06400-y)
Supplement: Supplementary file 1 — Supplementary Information [file 41598_2017_6400_MOESM1_ESM.pdf]

Efficient increase of γ-aminobutyric acid (GABA) content in tomato fruits by targeted mutagenesis

Author initials and surname

Satoko Nonaka[1,2], Chikako Arai[3], Mariko Takayama[1], Chiaki Matsukura[1,2] and Hiroshi Ezura[1,2*]

1: Faculty of Life and Environmental Sciences, University of Tsukuba, 1-1-1 Tennodai, Tsukuba, Ibaraki 305-8572, Japan

2: Gene Research Center, University of Tsukuba, 1-1-1 Tennodai, Tsukuba, Ibaraki 305-8572, Japan

3: Graduate School of Life and Environmental Sciences, University of Tsukuba, 1-1-1 Tennodai, Tsukuba, Ibaraki 305-8572, Japan

[*] Correspondence to E-mail: ezura.hiroshi.fa@u.tsukuba.ac.jp

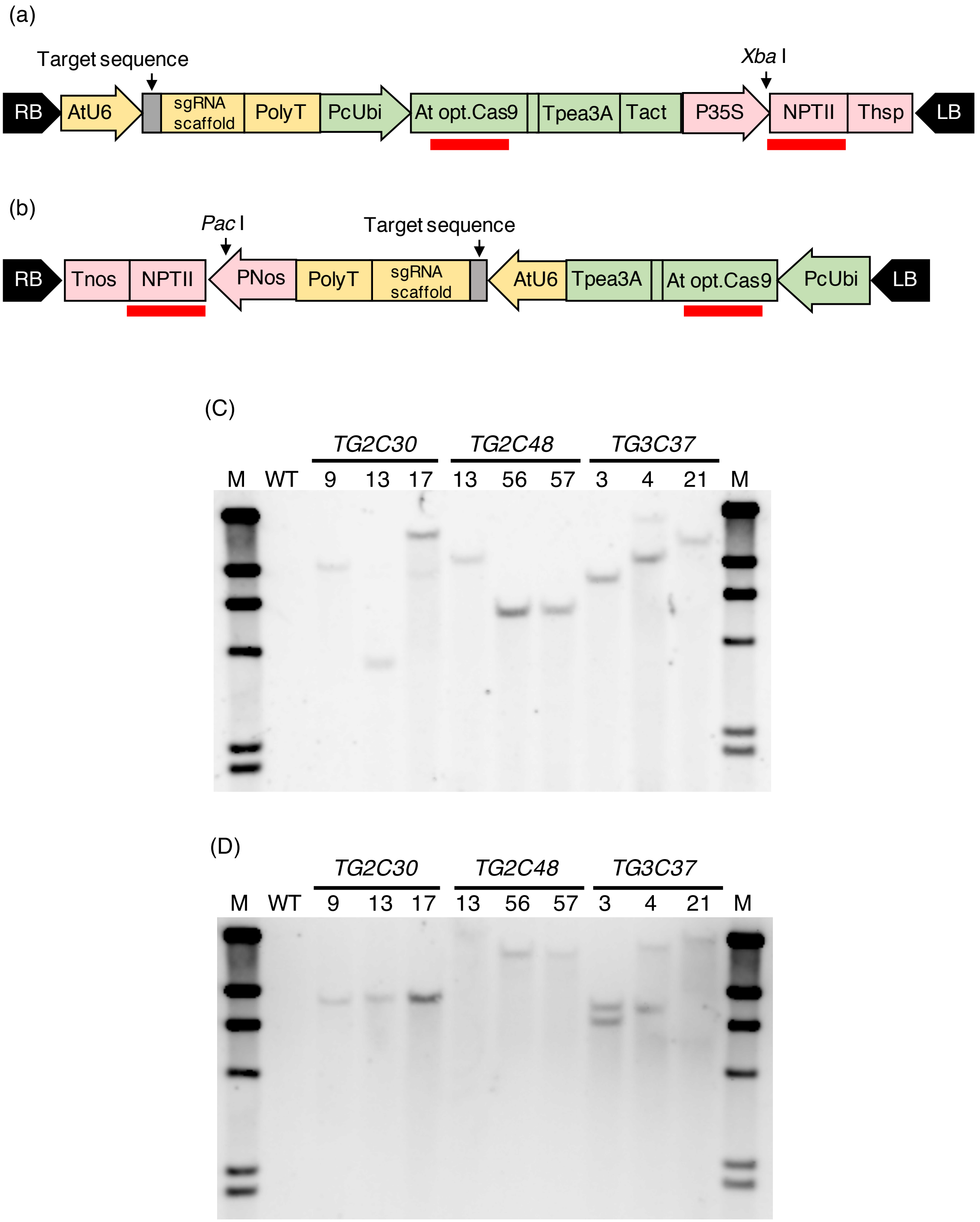

Supplementary Figure 1. C-terminals detected in the T_0 generation. The Types of C-terminal detected in this study were classified into four groups. The putative zygosity of the T_0 generation was homozygous, heterozygous, bi-allelic, or chimera. The green box indicates the C-terminal of SlGADs, including the autoinhibitory domain. The blue box indicates truncated C-terminals and pink indicates SlGADs without a C-terminal. 'Met' and 'Stop' are the start and stop codons, respectively.

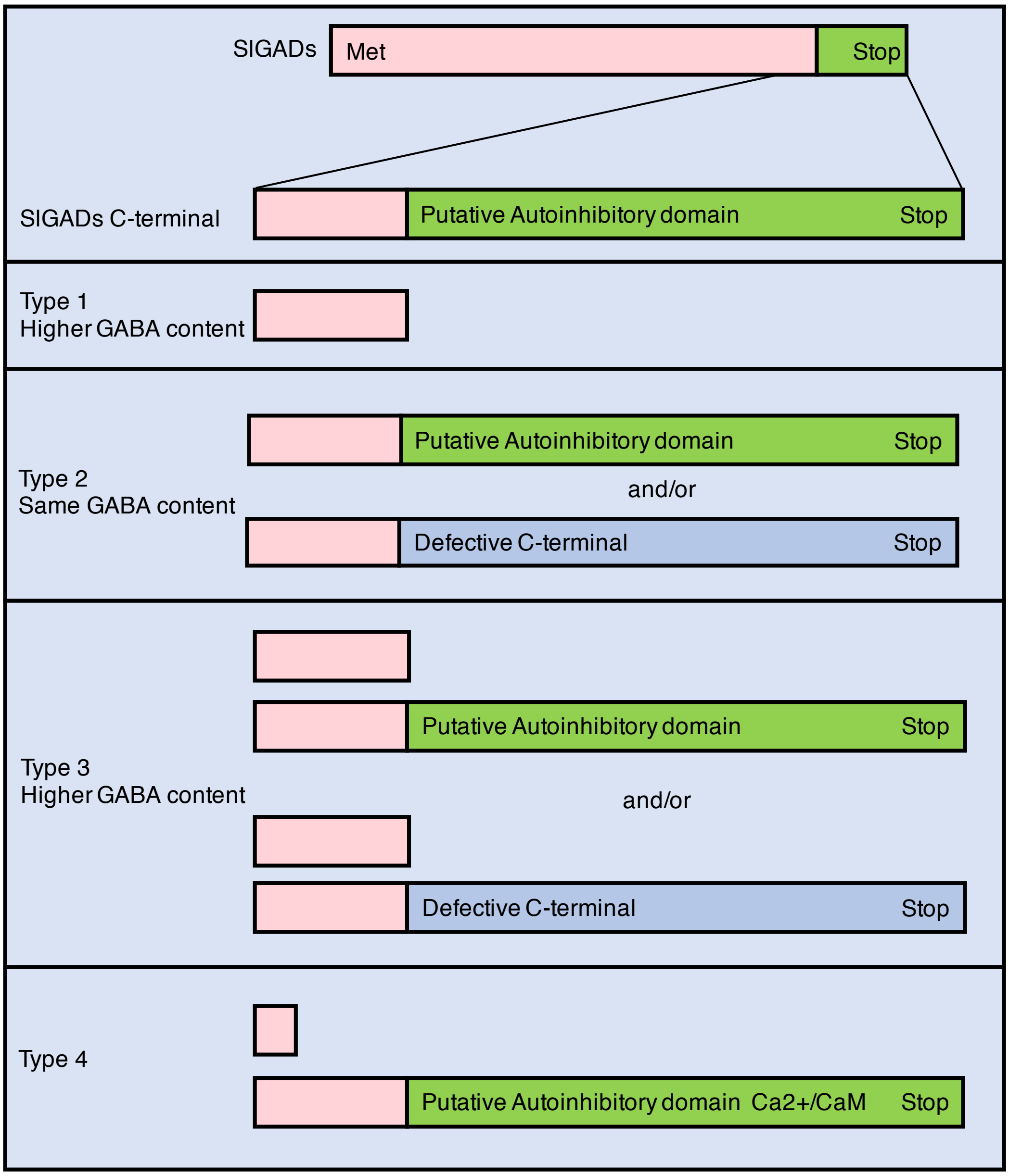

Supplementary Figure 2. Map of the CRISPR/Cas9 vector and the southern blot analysis of the T_0 generation. (**a**) and (**b**) present the maps of the T-DNA region in the CRISPR/Cas9 expression vectors used for TG2C30 and for TG2C48 and TG3C37, respectively. (**a**) and (**b**) were provided by Prof. Puchta Holger (Botanical Institute II, Karlsruhe Institute of Technology, Karlsruhe, Germany) and Prof. Seiichi Toki (NARO, Japan), respectively. Red bars represent the position of probes used in southern blot analysis. *Xba*I and *Pac*I indicate restriction enzyme sites that were used in southern hybridization. NPTII and Cas9 probes were used in (**c**) and (**d**). M indicates the marker λ/*Hind*III. WT indicates the genome of wild type tomato, used as the negative control.

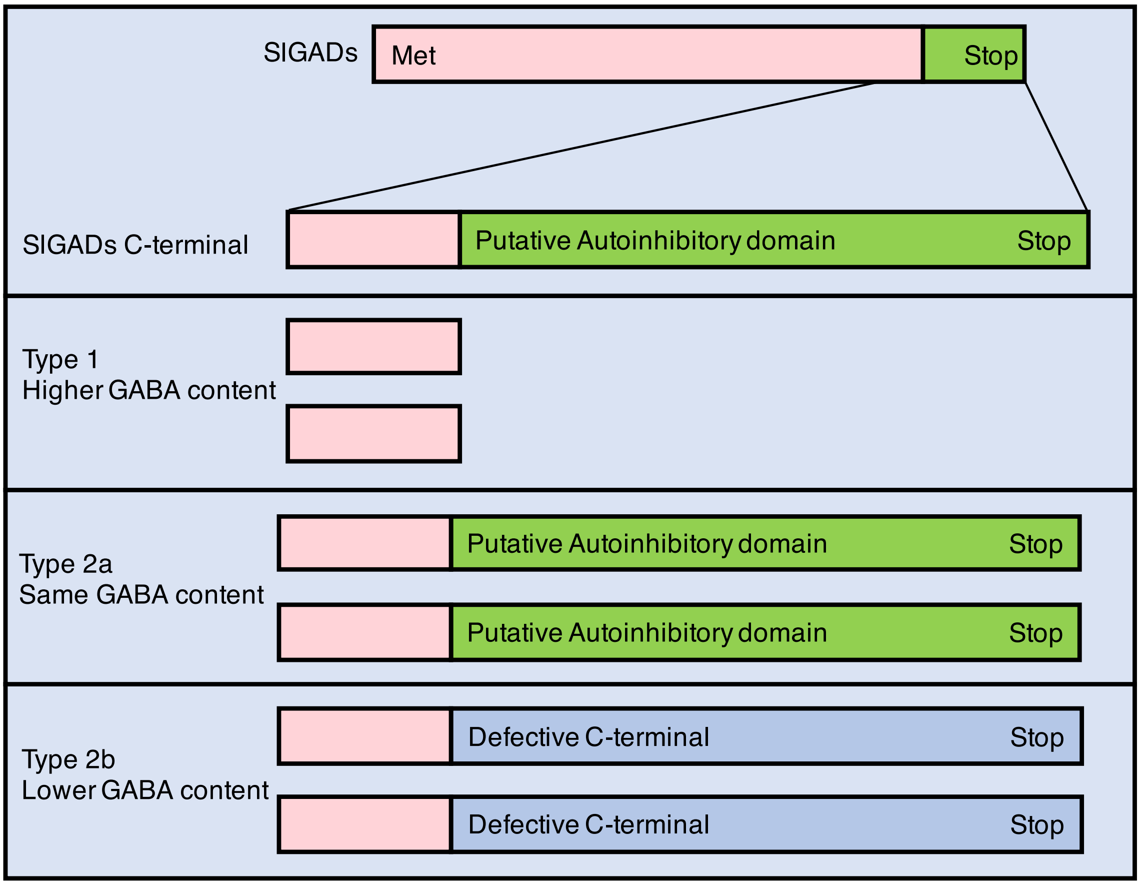

Supplementary Figure 3. Structure of the C-terminal in the T_1 generation. The C-terminal of SlGADs in the T_1 generation were classified into three types. All plants were homozygous. The green, blue, and pink boxes indicate the C-terminal of SlGADs including the autoinhibitory domain, truncated C-terminal, and SlGADs without a C-terminal, respectively. 'Met' and 'Stop' are the start and stop codons, respectively.

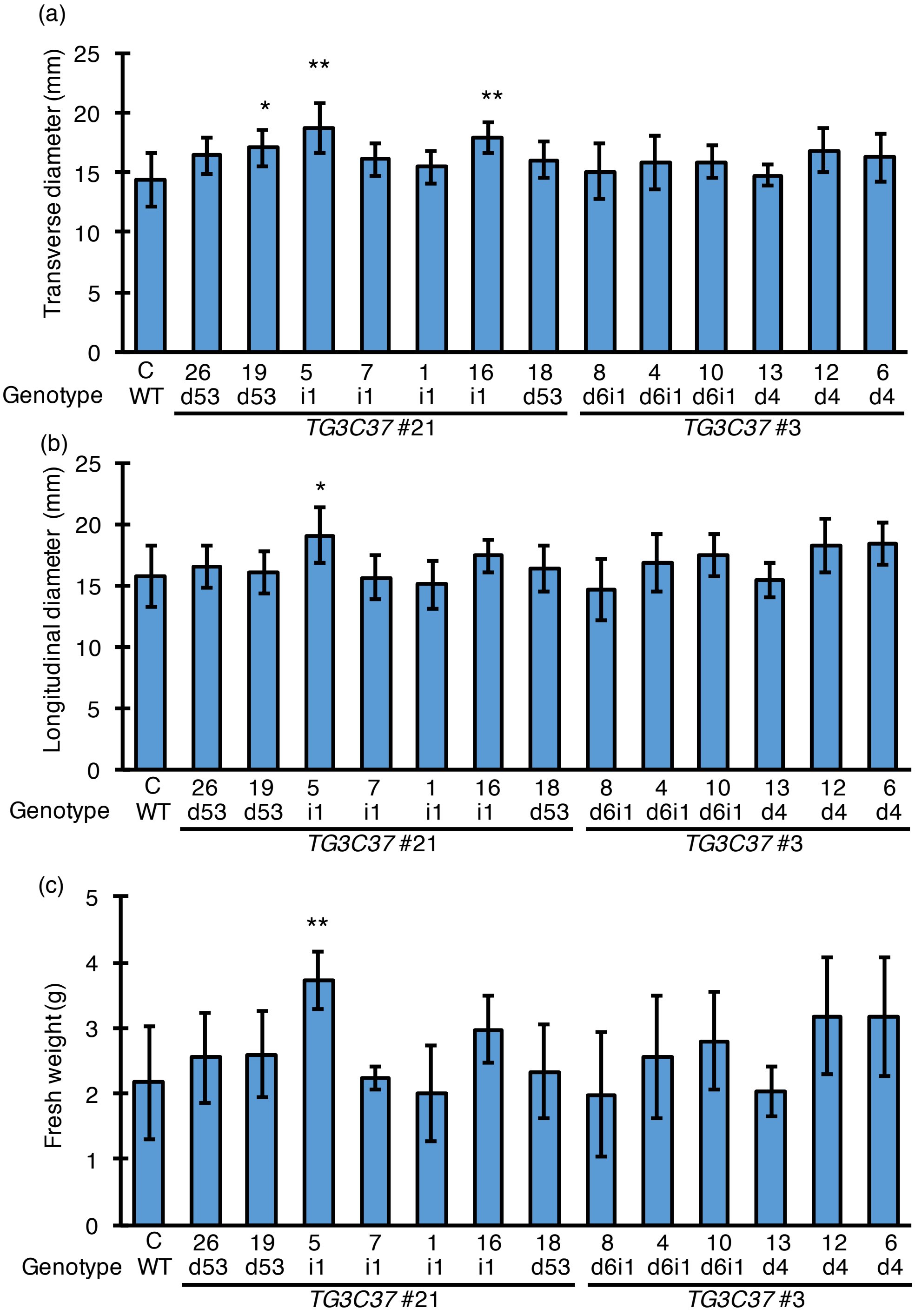

Supplemental figure 4. Fruit size of red stage in T_1 generation. (a), (b), and (c) indicaited transverse diameter, longitudinal diameter and fresh weight of red stage (Breaker + 10 days) fruit. Bars indicate standard deviation (n = 3) and asterisks indicate statistical differences in relation to control according to the Tukey-Kramer's test (*P <0.05 and **P <0.01). 'C' is the control plant with WT genotype.

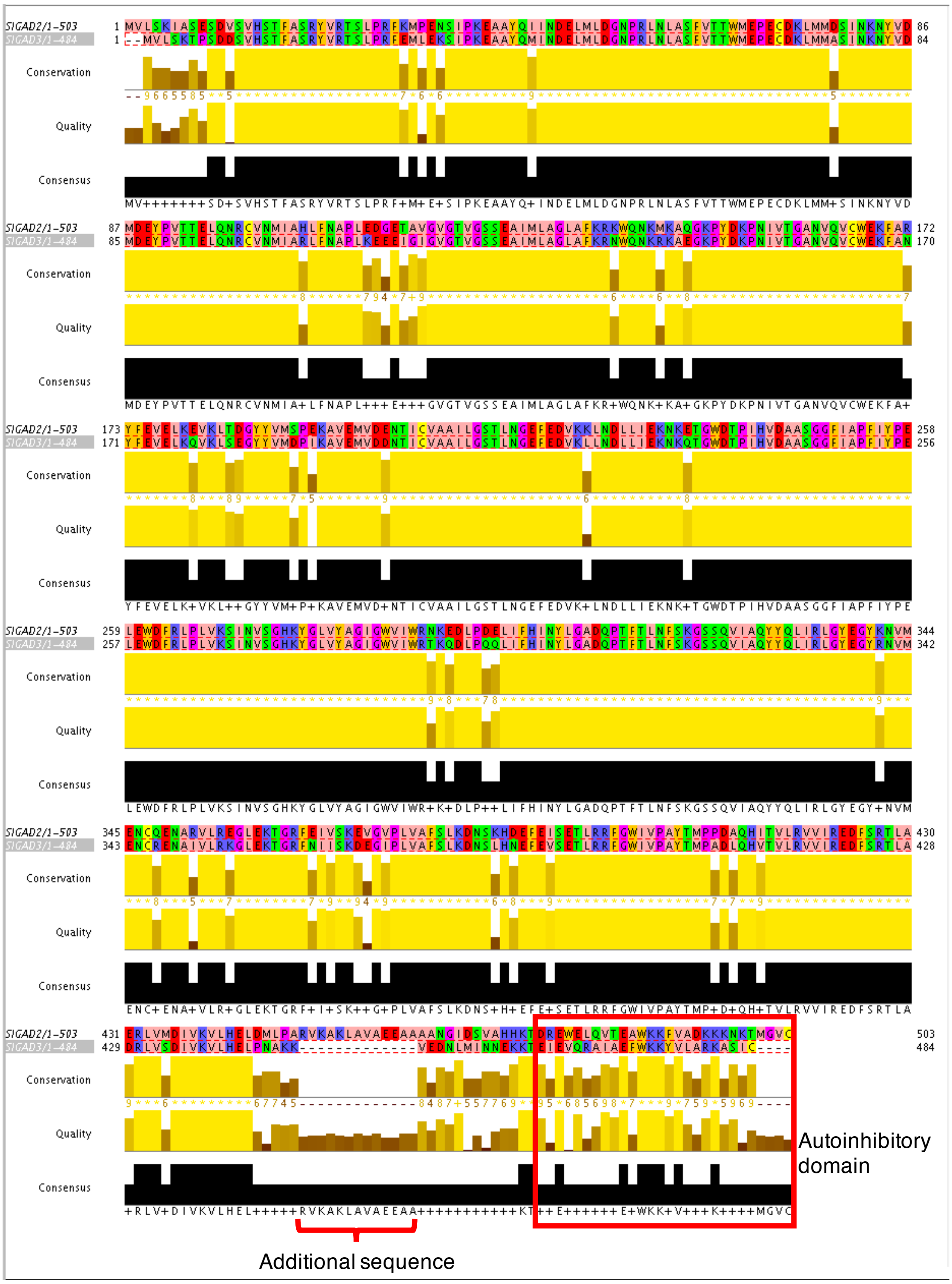

Supplementary Figure 5. Amino acid sequences of SlGAD2 and SlGAD3. The multiple alignment of the amino acid sequences was performed in ClustalW (http://www.genome.jp/tools/clustalw/), and the arrangement was displayed using the Jalview program (http://www.jalview.org). Amino acids were coloured according to Zappo in Jalview. Conservation, Quality, and Consensus were also calculated in Jalview.

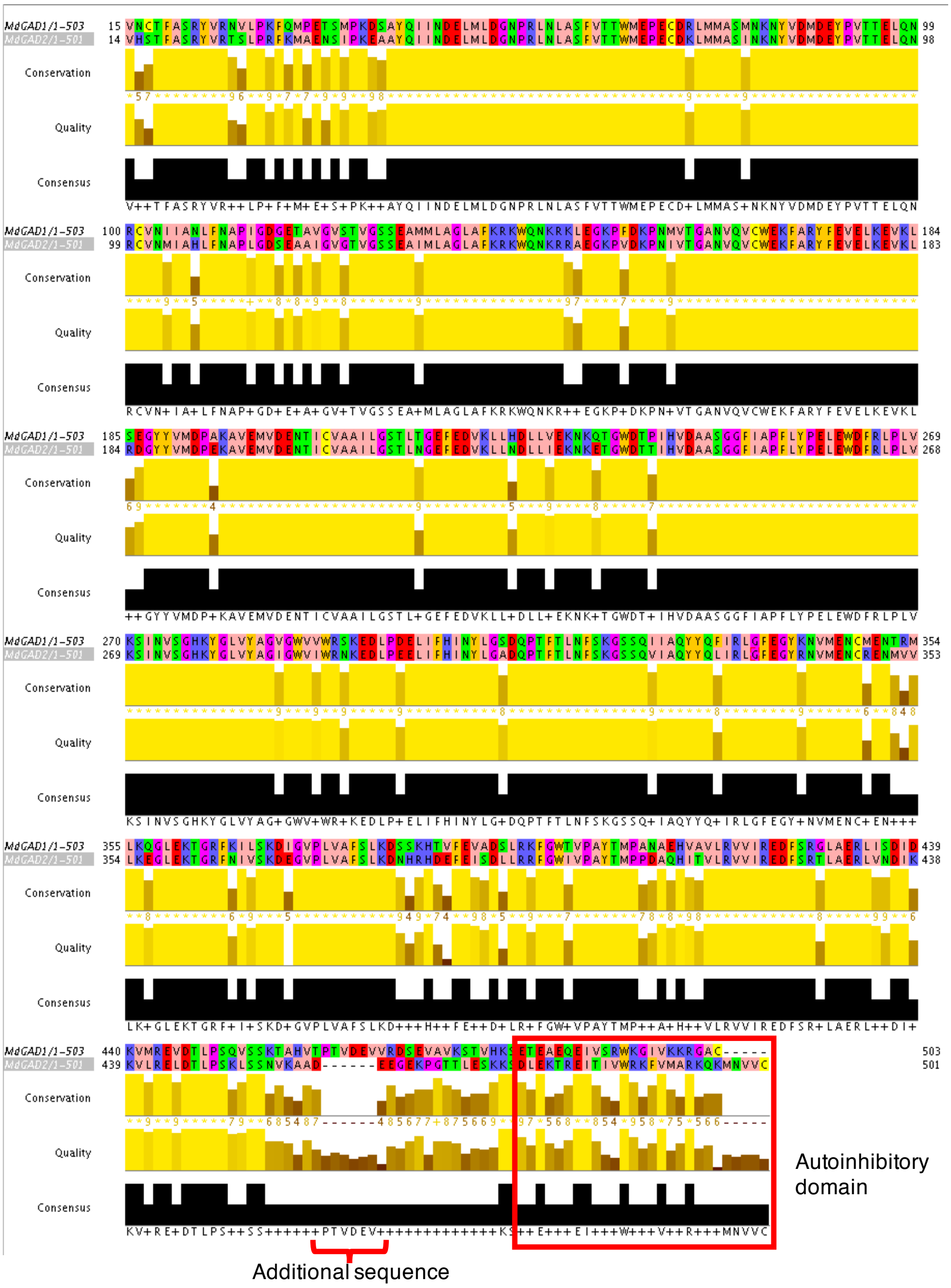

Supplementary Figure 6. Amino acid sequences of MdGAD1 and MdGAD2. The multiple alignment of the amino acid sequence of apple MdGAD1 and MdGAD2, which show calmodulin-dependent activity, was performed in ClustalW (http://www.genome.jp/tools/clustalw/), and the arrangement was displayed using the Jalview program (http://www.jalview.org). Amino acids were coloured according to Zappo in Jalview. Conservation, Quality, and Consensus were also calculated in Jalview.

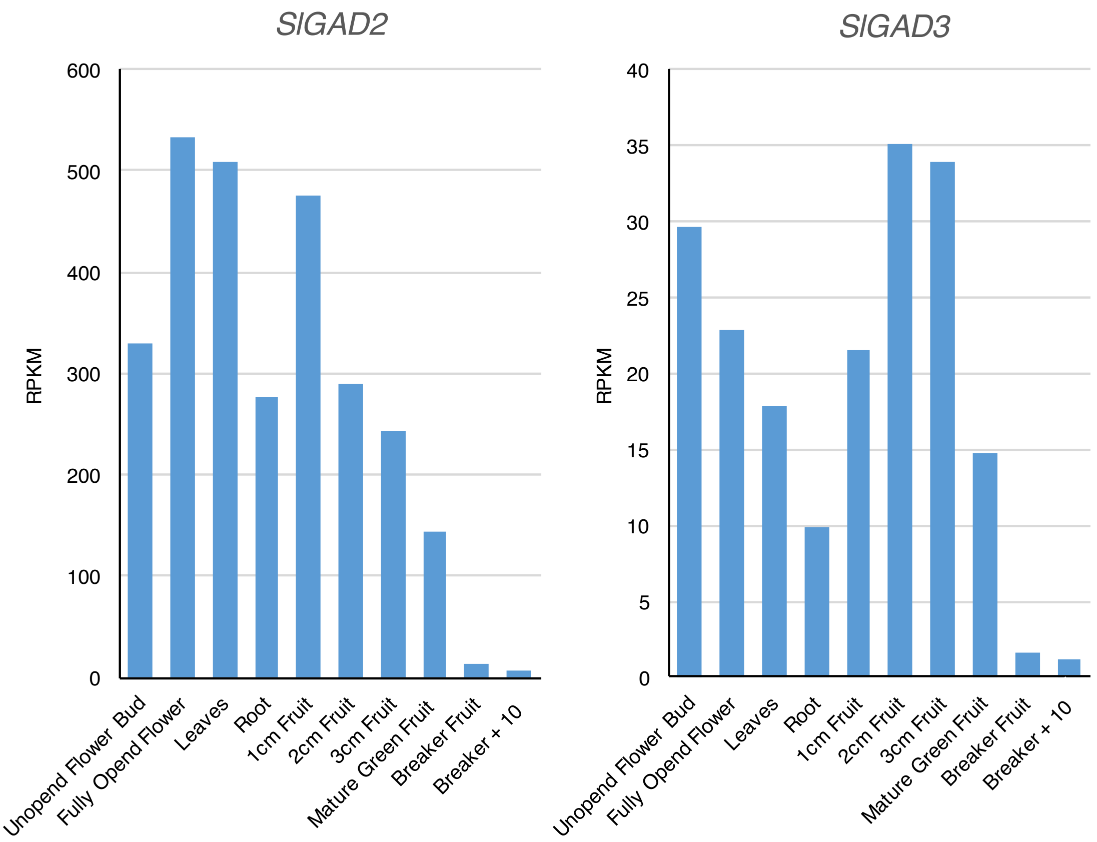

Supplementary Figure 7. Expression patterns of *SlGAD2* and *SlGAD3* in tomato. Dataset from the Tomato eFP browser (http://www.bar.utoronto.ca/efp_tomato/cgi-bin/efpWeb.cgi). *GAD2* expression was about 10 times higher than *GAD3* expression. Leaves showed the same level of *GAD2* expression as 1 cm fruits and these were the highest values. Fruits with 2 and 3 cm presented the highest *GAD3* expression, which was twice higher than that in leaves.
